# Mapping genomic regions of moisture deficit stress tolerance using backcross inbred lines in wheat (*Triticum aestivum* L.)

**DOI:** 10.1038/s41598-020-78671-x

**Published:** 2020-12-10

**Authors:** Shashikumara Puttamadanayaka, Harikrishna, Manu Balaramaiah, Sunil Biradar, Sunilkumar V. Parmeshwarappa, Nivedita Sinha, S. V. Sai Prasad, P. C. Mishra, Neelu Jain, Pradeep Kumar Singh, Gyanendra Pratap Singh, Kumble Vinod Prabhu

**Affiliations:** 1 ICAR-Indian Agricultural Research Institute, New Delhi, 110 012 India; 2 ICAR-Indian Wheat and Barley Research Institute, Karnal, India; 3 PPV & FR Authority NAAS Complex, New Delhi, 110012 India; 4 ICAR-Indian Grassland and Fodder Research Institute, Jhansi, 284003 India; 5 Jawaharlal Nehru Krishi Vishwa Vidyalaya, Jabalpur, Madhya Pradesh 482 004 India

**Keywords:** Biotechnology, Genetics

## Abstract

Identification of markers associated with major physiological and yield component traits under moisture deficit stress conditions in preferred donor lines paves the way for marker-assisted selection (MAS). In the present study, a set of 183 backcross inbred lines (BILs) derived from the cross HD2733/2*C306 were genotyped using 35K Axiom genotyping array and SSR markers. The multi-trait, multi-location field phenotyping of BILs was done at three locations covering two major wheat growing zones of India, north-western plains zone (NWPZ) and central zone (CZ) under varying moisture regimes. A linkage map was constructed using 705 SNPs and 86 SSR polymorphic markers. A total of 43 genomic regions and QTL × QTL epistatic interactions were identified for 14 physiological and yield component traits, including NDVI, chlorophyll content, CT, CL, PH, GWPS, TGW and GY. Chromosomes 2A, 5D, 5A and 4B harbors greater number of QTLs for these traits. Seven Stable QTLs were identified across environment for DH (*QDh.iari_6D*), GWPS (*QGWPS.iari_5B*), PH (*QPh.iari_4B-2, QPh.iari_4B-3*) and NDVI (*QNdvi1.iari_5D*, *QNdvi3.iari_5A*). Nine genomic regions identified carrying major QTLs for CL, NDVI, RWC, FLA, PH, TGW and biomass explaining 10.32–28.35% of the phenotypic variance. The co-segregation of QTLs of physiological traits with yield component traits indicate the pleiotropic effects and their usefulness in the breeding programme. Our findings will be useful in dissecting genetic nature and marker-assisted selection for moisture deficit stress tolerance in wheat.

## Introduction

Drought critically affects wheat (*Triticum aestivum* L.) crop growth and productivity, causing a decrease in yield by 27.5%^1^. Depletion of water resources and drastic climate change challenge wheat production. Globally arid and semi-arid regions contribute about 60% of crop production. The rainfall pattern critically fluctuates in these areas creating drought stress, which is one of the most common causes for yield loss. Wheat being one of the major cereal crops is grown worldwide in an area of 218.54 million hectare with a world production of 771.71 million tons^2^. But, nearly 50% of the world wheat growing area (230 million hectares) is affected by moisture deficit stress^3^.

The observed phenotype of moisture deficit stress tolerance in plants is a product of quantitatively expressing genes and their interaction with temperature and moisture content in the environment^4^. Tackling these traits through traditional breeding progenies can be difficult due to gene × environment interaction in large segregating heterogeneous populations. The DNA markers linked or associated with these genomic regions harboring the quantitative trait loci (QTL) responsible for trait variation have however, proved to improve precision in identifying the desired plants with maximized genetic gain in selection^5^. Hence, QTL mapping is an important prerequisite for locating the QTLs responsible for the observed phenotypic variation in physiological traits and yield components under moisture stress^4^. The difficulty in phenotyping physiological traits in a large population can be overcome by adopting of normalized difference vegetative index (NDVI), canopy temperature (CT), relative water content (RWC) and chlorophyll content measured by SPAD chlorophyll meter as they are robust and have high potential in screening the genotypes under moisture stress condition^6^. Despite the availability of these advanced tools for phenotyping of physiological traits, so far, only a few studies have been able to report validated QTLs for physiological traits^7–9^. However, the reported studies were conducted under controlled greenhouse conditions or growth chambers which are yet to be translated under field conditions in the targeted environments where the plants are exposed to far more factors than those fixed under controlled conditions. The QTL, detected under controlled conditions could behave differently when challenged under field situations related to the trait expression^10^. Thus, characterizing the population under field-based moisture deficit conditions in the target environment will be the most realistic basis for identifying QTLs and their contribution to trait expression.

Achieving high grain yield (GY) under water limited condition is a difficult exercise because of the complex nature of yield as a trait due to the variable responses and interactions among its component traits like thousand grain weight (TGW), grain weight per spike (GWPS) and harvest index (HI) under different physiological regimes the crop is exposed to. Thus, seeking genetic components that result in the improvement of yield component traits in combination with the targeted physiological environment which in turn will result in improved yield appears to be more predictable approach for the target environment than directly looking for the loci controlling grain yield itself in the same situation. The allelic combinations of QTLs along with their mode of action responsible for the desired physiological and yield component trait expressions in a genotype are expected to result in increased GY under moisture deficit stress conditions in a predictable manner to plan a breeding programme involving the chosen parental genotypes. Many attempts have been made to map genomic regions for plant height (PH), coleoptile length (CL), TGW, GY and GWPS^11–15^. Parallel studies on deciphering the genomic regions affecting the various physiological and yield component traits will serve as robust resources for breeding superior drought tolerant varieties in wheat for similar target environments.

Identification of desirable donors for physiological traits responsible for moisture deficit stress tolerance and yield components under moisture stress, as well as the availability of suitable mapping populations with such donors as parents is a prerequisite for QTL mapping. In wheat, QTL mapping for moisture deficit stress reported so far has employed biparental mapping populations^16–19^. However, though the usefulness of backcross inbred line (BILs) method in breeding was well described^20^, there are very few reports on QTL mapping using backcrossed inbred lines in wheat or other crops^21^. The immortality of BILs developed as introgressed lines for targeted traits only, gives BILs an enormous focus for testing only for these traits in a large number of replicated trials over the target environment locations with scope to effect independent simultaneous selection of lines fixed for desired donor’s alleles of QTLs. BILs without any imposed selection would segregate in the ratio of 3:1 between the recipient and donor parents, and therefore, they possess a low proportion of the donor parent in each genotype making recovery of recipient genotype for the non-targeted genomic regions which do not harbor the trait specific QTL alleles from the donor parent in the progeny lines, making the population ideally suited for mapping specific genomic regions responsible for the desired QTL variation. The involvement of large genome size (~ 17 Gb) composed of three basic genomes sharing homology amongst confounded by the relatively low level of polymorphism for DNA markers between wheat varieties have, in general, hampered the development of saturated genetic linkage maps in wheat^22^. This situation can be overcome by the use of SNP genotyping platforms and the availability of wheat genome sequence data. The large number of SNP with an allelic distinction between source parents in the progenies and codominant nature has enabled the development of a saturated linkage map in wheat. These saturated linkage maps improve the efficiency of revealing genomic regions underlying the complex economic important traits^23^.

In the present study, we developed a BIL population of 183 BC_1_F_6_ lines derived from an elite cross between HD2733(ATTILA /3/TUI /CARC //CHEN / CHTO /4/ATTILA) and a well-adapted drought tolerant variety C306(RGN/CSK3 //2* C591/3/C217/N14 //C281) in central India and identified as a donor for drought tolerance. Variety C306 continues to be grown widely under warm unirrigated conditions for more than seven decades and continues to be a standard check for moisture deficit stress tolerance in all wheat breeding programmes for drought tolerance in India. These two parents showed distinctive expression for component traits such as PH, GWPS, GY, TGW, CL, NDVI, chlorophyll content, RWC and CT. For genotyping, a 35K Axiom genotyping array chip along with polymorphic SSR markers was used. The objective of this study was to identify QTLs controlling physiological and yield component traits for moisture deficit stress tolerance.

## Results

### Multilocation phenotyping under different moisture availability situations

**Figure 1:**
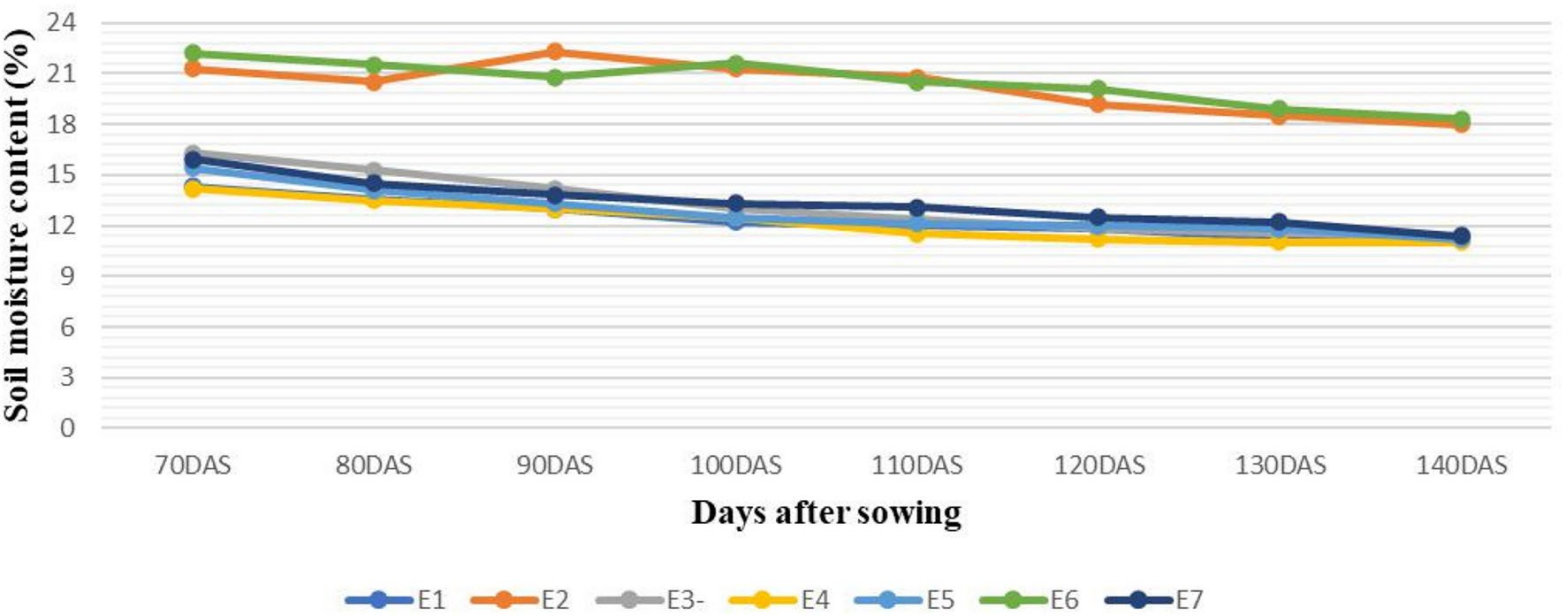
Periodical soil moisture content (%, w/w) under different crop growing environments.

The BIL population along with their parents were evaluated at Delhi and two drought prone locations in the central hot plains of India at Indore and Powarkheda (Table 1). The average soil moisture content (w/w) during the grain filling period was highest under irrigated condition (E1: 20.23%, E2: 20.48%) followed by restriction irrigation (E7: 13.34%, E3: 13.24%) and rainfed (E5:12.8%, E1: 12.41%, E4: 12.23%) condition (Fig. 1). There were 7 physiological traits considered here, of which NDVI was measured at 6 stages and CT measured at 4 stages followed by 7 yield related traits including biomass measured at post-flowering stages. The comparison between parents for their mean trait expression across seven environments for physiological traits, indicated higher trait values in C306 (P2) than HD2733 (P1), for chlorophyll content (P1: 48.97, P2:50.65), RWC (P1:63.33, P2:72.42), CL (P1:5.67 cm, P2:9.94 cm), NDVI (P1:0.51, P2:0.52) and lower trait value for CT (P1:24.05: P2:22.98). Similarly, donor parent C306 (P2) consistently showed average higher trait values than HD2733 (P1), for GY (P1:324 g, P2:457.5 g), GWPS (P1:16.56 g, P2:20.26 g), TGW (P1:45.66 g, P2:53.14 g), PH (P1:84.4 cm, P2: 117.10 cm), biomass (P1:901.5 g, P2:1336 g), AL (P1:4.56 cm, P2:7.76 cm) and FLA (P1:23.36 cm^2^, P2:26.42 cm^2^), across all locations under unirrigated conditions (Supplementary Table S1 and Supplementary Table S2). Among physiological traits, the average *H*^2^ estimate across all environments for NDVI was 0.63 and for CT was 0.57. The trait range for NDVI varied from 0.09 (NDVI-6, in E1) to 0.83 (NDVI-3, in E3). The average *H*^2^ for SPAD was 0.59 and the range for trait value varied from 25.9 to 63.4 in E3 environment. Further, coleoptile length range was from 4.60 to 12.64 cm in BILs with a heritability estimate of 0.75. The highest TGW (69.5 g) was observed in E2 and the lowest was in E6 (27.55) with an average *H*^2^ estimate of 0.67. The *H*^2^ estimates across environments for GY, GWPS, HI, PH and biomass were 0.73, 0.71, 0.73, 0.67 and 0.76, respectively. The mean values across environments were 417.29 g/plot, 18.54 g/plot, 0.35, 105.65 cm 1172.08 g/plot for GY, GWPS, HI, PH and biomass, respectively (Supplementary Table S1 and Supplementary S2). A significant positive correlation was observed for physiological traits NDVI-2, NDVI-3, NDVI-4, NDVI-5, RWC and SPAD with GY. CT in vegetative and reproductive stage negatively associated with GY, NDVI-2, NDVI-4, NDVI-5, GWPS, TGW and SPAD. The major agronomic traits GWPS, TGW, Biomass were positively correlated with yield (supplementary Table S3). In general, the BIL population was characterized by a significant range of variability with transgressive range of phenotypic values in several lines for each trait observed (Supplementary Table S4a,b).

**Table 1:** Characteristics of the studied environments, period of the experiments in each field trial.

Growing season	Location	Code	Latitude	Longitude	Altitude (m)	Sowing date	Harvesting date
2016–2017	Delhi rainfed	E1	28° 40′ N	77° 13′ E	228	09-11-2016	10-04-2017
2016–2017	Delhi irrigated	E2	28° 40′ N	77° 13′ E	228	10-11-2016	12-04-2017
2016–2017	Indore restricted irrigation	E3	22° N′	75° E	553	29-10-2016	20-03-2017
2016–2017	Powarkheda rainfed	E4	22° N′	77° E	308	28-10-2016	24-03-2017
2017–2018	Delhi rainfed	E5	28° 40′ N	77° 13′ E	228	08-11-2017	11-04-2018
2017–2018	Delhi irrigated	E6	28° 40′ N	77° 13′ E	228	09-11-2017	13-04-2018
2017–2018	Indore restricted irrigation	E7	22° N′	75° E	553	27-10-2017	20-03-2018

### Construction of linkage map

705 of the 35K Axiom SNP array along with 86 SSR marker loci out of 728 markers identified as polymorphic on 21 wheat chromosomes are used for the construction of genetic linkage map (Table 2). Graphical analysis of genotypes indicated monogenic mendelian segregation between HD2733 (67.5%) and C306 (22%) alleles in the population, respectively with residual heterozygosity prevalent in 10.5% loci combined with missing data (Supplementary Fig. S1). The total linkage map spanned over 4700.15 cM, ranging from 2.78 cM/marker for 1A to 21.75 cM/marker for 5D chromosome with an average marker density of 5.93 cM/marker (Supplementary Table S5). Altogether, the number of markers mapped on the B genome (358) was higher compared to the A genome (334) and considerably fewer markers mapped on the D genome (99).

**Table 2:** QTLs detected for various physiological and yield component traits in the HD2733/2*C306 backcross inbred lines population.

Trait	Location	QTL	Position	Marker interval	LOD	PVE (%)	Additive effect	Confidence interval (cM)
CL	E1	*QCl.iari_4B*	96	AX-94496390-AX-94957045	11.24	28.35	− 0.83	94.5–98.5
	E1	*QCl. iari_5D*	314	AX-94666462-AX-95248961	3.32	12.63	− 0.74	304.5–323.5
NDVI-1	E2	*QNdvi1.iari_5D*	282	Xwmc318-AX-94666462	3.65	7.40	0.074	274.5–288.5
NDVI-2	E1	*QNdvi2.iari_2A*	334	AX-94497666-AX-94569814	4.06	11.20	0.025	328.5–336.5
	E6	*QNdvi2.iari_6D*	182	AX-95006509-Xbarc54.1	2.98	9.11	0.027	172.5–191.5
	E3	*QNdvi2.iari_5D*	283	Xwmc318-AX-94666462	3.03	5.71	0.019	277.5–291.5
	E4	*QNdvi2.iari_5A*	240	AX-95001743-AX-95152679	3.69	8.13	− 0.02	238.5–244.5
	E4	*QNdvi2.iari_6B*	77	AX-94523287-AX-94610216	4.47	9.71	− 0.04	73.5–87.5
NDVI-3	E2	*QNdvi3.iari_5A*	221	AX-94414339-AX-94730618	2.67	4.58	− 0.016	218.5–222.5
	E2	*QNdvi3.iari_5D*	71	Xgdm63-Xcfd29	3.14	8.62	− 0.023	62.5–77.5
	E7	*QNdvi3.iari_2D*	44	AX-94429402-AX-95164994	2.61	5.12	− 0.028	40.5–48.5
	E7	*QNdvi3.iari_6A*	17	AX-94407050-Xwmc417	3.62	9.46	− 0.032	15.5–24.5
	E2	*QNdvi3.iari_2A*	280	AX-95211689-AX-95193898	2.71	8.06	0.010	278.5–286.5
NDVI-4	E1	*QNdvi4.iari_5A*	221	AX-94414339-AX-94730618	3.30	8.08	− 0.028	218.5–222.5
NDVI-5	E1	*QNdvi5.iari_6D*	97	Xbarc183-AX-95010178	3.78	9.30	− 0.039	85.5–98.5
	E5	*QNdvi5.iari_6A*	101	AX-95247279-AX-94870783	3.51	8.55	0.037	96.5–108.5
NDVI-6	E1	*QNdvi6.iari_7A*	144	AX-94976788-AX-94756419	2.70	6.26	− 0.036	142.5–145.5
	E6	*QNdvi6.iari_2A*	170	Xgdm93-AX-94389568	2.87	6.26	− 0.05	167.5–172.5
	E6	*QNdvi6.iari_7B*	132	Xwmc517-Xwmc501	8.64	7.21	− 0.07	125.5–138.5
Chl. content	E1	*QChl.iari_5A*	221	AX-94414339-AX-94730618	4.81	9.30	1.09	218.5–221.5
	E1	*QChl.iari_5B*	33	AX-95091073-AX-94525037	4.25	8.27	1.11	31.5–36.5
	E1	*QChl.iari_2A*	61	AX-94465394- AX-94876482	3.57	6.75	− 0.84	58.5–63.5
	E5	*QChl.iari_6D*	183	AX-95006509-Xbarc54.1	2.67	2.10	4.86	175.5–189.5
	E2	*QChl.iari_4A*	207	AX-94592862- AX-94460475	3.12	7.59	2.42	197.5–212.5
	E6	*QChl.iari_5A*	230	AX-94531685-AX-94726381	3.52	8.34	0.68	228.5–230.5
	E6	*QChl.iari_3B*	207	Xwmc689-Xwmc78	3.44	8.61	− 1.04	195.5–217.5
	E3	*QChl.iari_5D*	313	AX-94666462-AX-95248961	2.62	8.10	3.85	304.5–326
CT-1	E2	*QCt1.iari_2A*	324	AX-94456186-Xgwm275	2.91	7.45	− 0.56	322.5–324.5
	E2	*QCt1.iari_7D*	31	Xwmc606.1-AX-95154446	3.38	9.26	0.57	23.5–34.5
CT-3	E3	*QCt3.iari_2A*	335	AX-94497666-AX-94569814	2.62	7.37	0.041	328.5–340.5
CT-4	E1	*QCt4.iari_5A*	106	Xgwm304-AX-94920631	4.07	9.09	0.59	90.5–115.5
RWC	E1	*QRwc.iari_5D*	321	AX-94666462-AX-95248961	2.51	5.40	2.02	306.5–326
	E1	*QRwc.iari_7A*	143	AX-94976788-AX-94756419	5.11	10.77	− 2.98	142.5–144.5
FLA	E1	*QFla.iari_2D*	23	AX-95151743-AX-94877462	3.72	11.44	− 2.91	15.5–35.5
	E1	*QFla.iari_6B*	240	XXwmc397-Xwmc494	3.06	3.69	1.69	232.5–245.5
AL	E1	*QAl.iari_4A*	31	Xbarc78-AX-94694992	2.99	4.30	− 0.16	21.5–39.5
PH	E1	*QPh.iari_4B-3*	107	AX-95630675-AX-94592612	4.38	9.31	− 2.64	99.5–111.5
	E2	*QPh.iari_4B-2*	95	AX-94496390-AX-94957045	8.36	13.92	− 4.62	94.5–97.5
	E5	*QPh.iari_4B-3*	104	AX-95630675-AX-94592612	5.57	13.67	− 3.746	99.5–110.5
	E3	*QPh.iari_7B*	266	AX-94622790-AX-95072534	3.68	7.34	4.44	262.5–268.5
	E7	*QPh.iari_4B-2*	95	AX-94496390-AX-94957045	3.56	8.48	− 3.30	93.5–96.5
TGW	E1	*QTgw.iari_7A*	135	AX-95097028-AX-94417618	3.55	9.91	− 7.59	133.5–135.5
	E2	*QTgw.iari_2B*	206	AX-95153769-Xbarc167	3.43	8.57	− 2.13	202.5–212.5
	E5	*QTgw.iari_2A*	84	AX-94995080-AX-94482613	3.06	11.20	3.2416	80.5–92.5
	E3	*QTgw.iari_5D*	87	Xcfd29-Xwmc215	2.64	7.11	2.05	70.5–94.5
GWPS	E5	*QGwps.iari_5B*	43	AX-94525037-AX-94755266	2.71	7.71	− 0.235	40.5–44.5
	E6	*QGwps.iari_1A*	23	AX-94901922-AX-94793862	3.97	8.61	0.87	22.5–24.5
	E6	*QGwps.iari_5B*	42	AX-94525037-AX-94755266	3.70	9.03	− 0.94	39.5–44.5
	E6	*QGwps.iari_7A*	227	Xwmc488.2-Xwmc606.2	3.15	7.60	− 0.85	214.5–238.5
GY	E2	*QYld.iari_2B*	155	AX-94493467-AX-94703320	2.79	7.14	− 38.58	150.5–156.5
	E5	*QYld.iari_2A*	43	AX-94698653-AX-94545499	3.62	7.67	− 21.71	40.5–46.5
	E5	*QYld.iari_6A*	122	AX-95247279-AX-94870783	3.42	7.91	− 23.44	109.5–125.5
HI	E1	*QHi.iari_2A*	56	AX-94867650-AX-95009146	4.18	7.67	− 0.02	55.5–57.5
	E1	*QHi.iari_5A*	229	AX-94531685-AX-94726381	4.28	8.31	0.02	224.5–230.5
	E1	*QHi.iari_2D*	44	AX-94429402-AX-95164994	3.17	6.52	0.02	38.5–48.5
	E3	*QHi.iari_5D*	86	Xcfd29-Xwmc215	2.79	6.00	0.02	78.5–100.5
	E7	*QHi.iari_5B*	193	AX-94655148-AX-95141741	3.59	8.20	0.03	190.5–196.5
DH	E6	*QDh.iari_6D*	183	AX-95006509-Xbarc54.1	3.10	6.23	5.06	169.5–193.5
	E1	*QDh.iari_6D*	196	AX-95006509-Xbarc54.1	2.76	6.99	4.73	162.5–203.5
BIOMASS	E2	*QBiomass.iari_7B*	126	Xwmc517-Xwmc501	2.85	10.32	181.84	110.5–136.5

Confidence interval (cM), Position of QTL located on chromosome: as cM distance from the top of each map.

LOD, A LOD threshold of 2.5 was used for declaration of QTL.

PVE (%), Phenotypic variance explained by QTL.

Additive effect, Positive “additive effect” indicates an increasing effect from HD2733; negative “additive effect” indicates an increasing effect from C306.

Location code; E1-Delhi rainfed (2016–2017), E2-Delhi irrigated (2016–2017), E3-Indore restricted irrigation (2016–2017), E4-Powarkheda rainfed (2016–2017), E5-(Delhi rainfed (2017–2018), E6-Delhi irrigated (2017–2018) and E7-Indore restricted irrigation (2017–2018).

**Figure 2:**
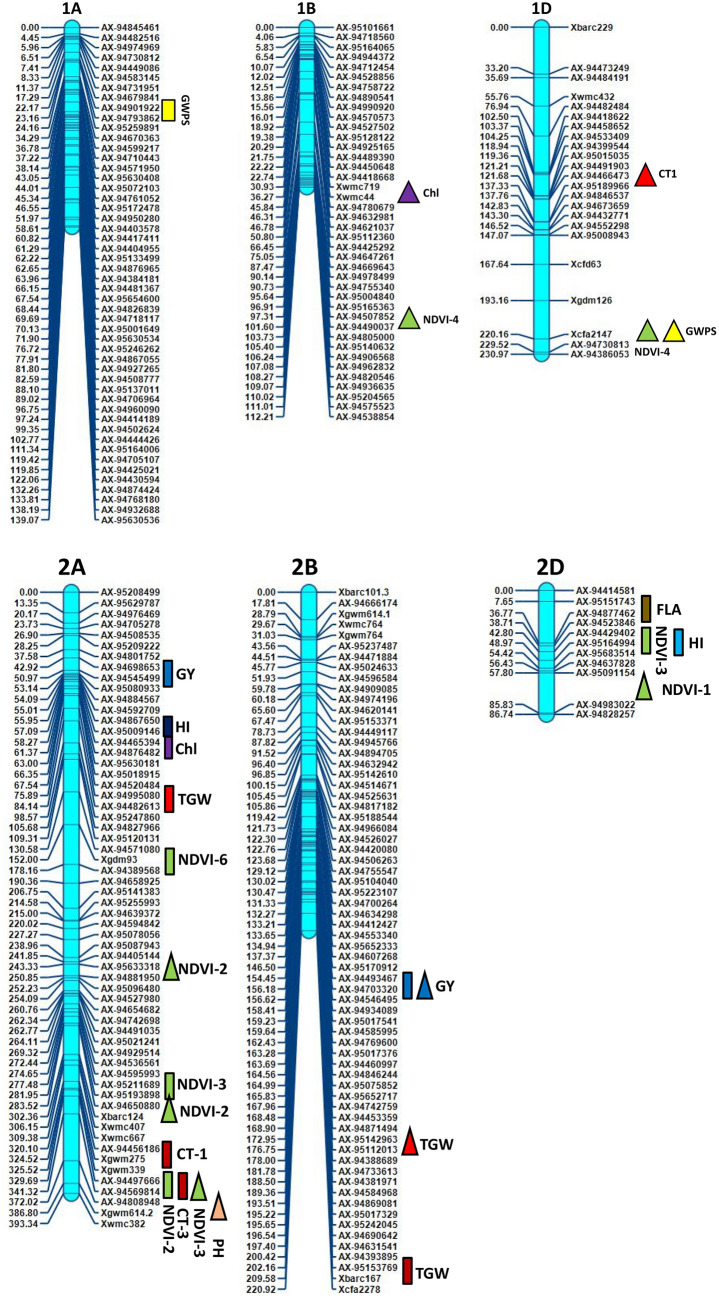

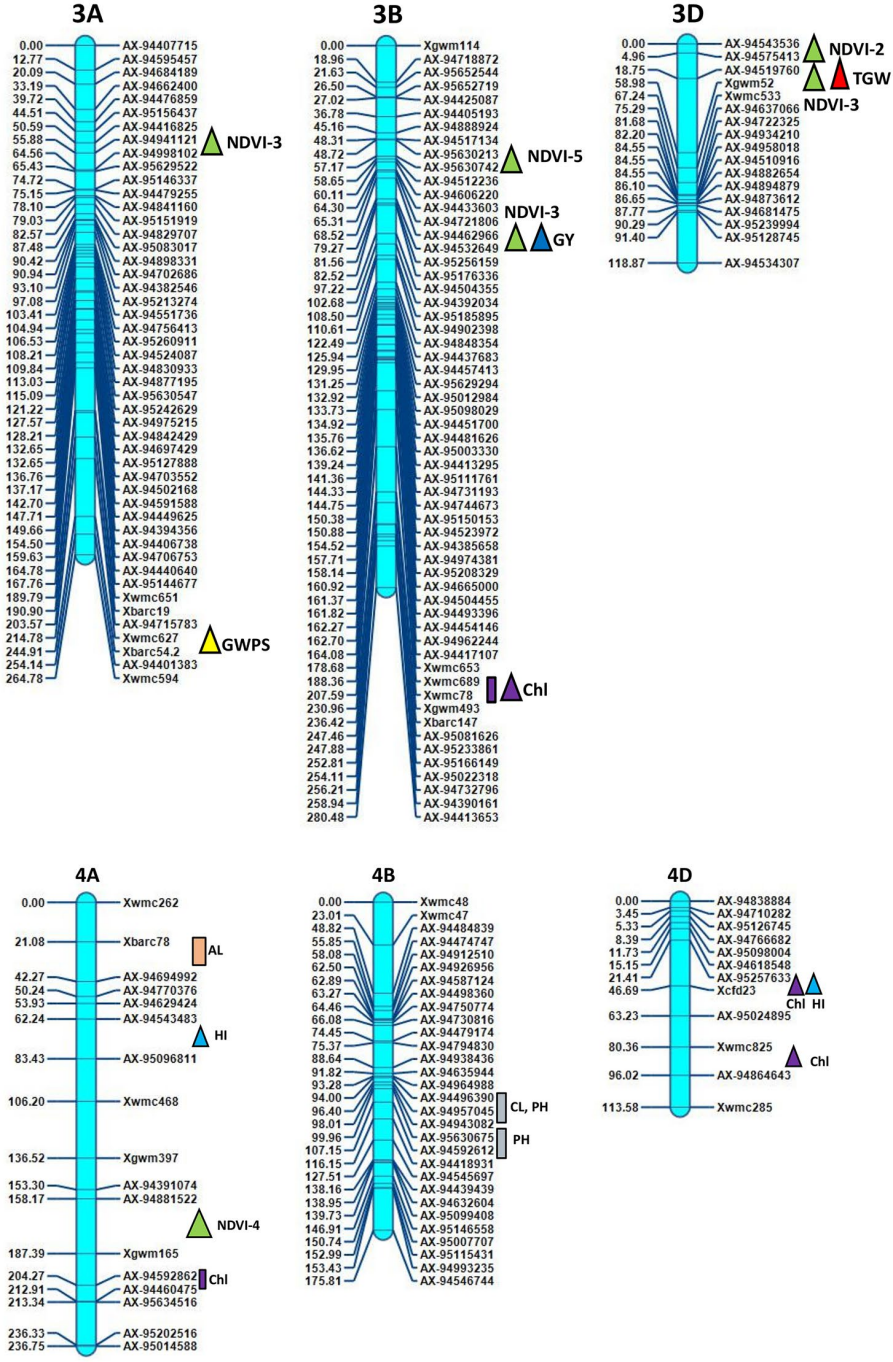

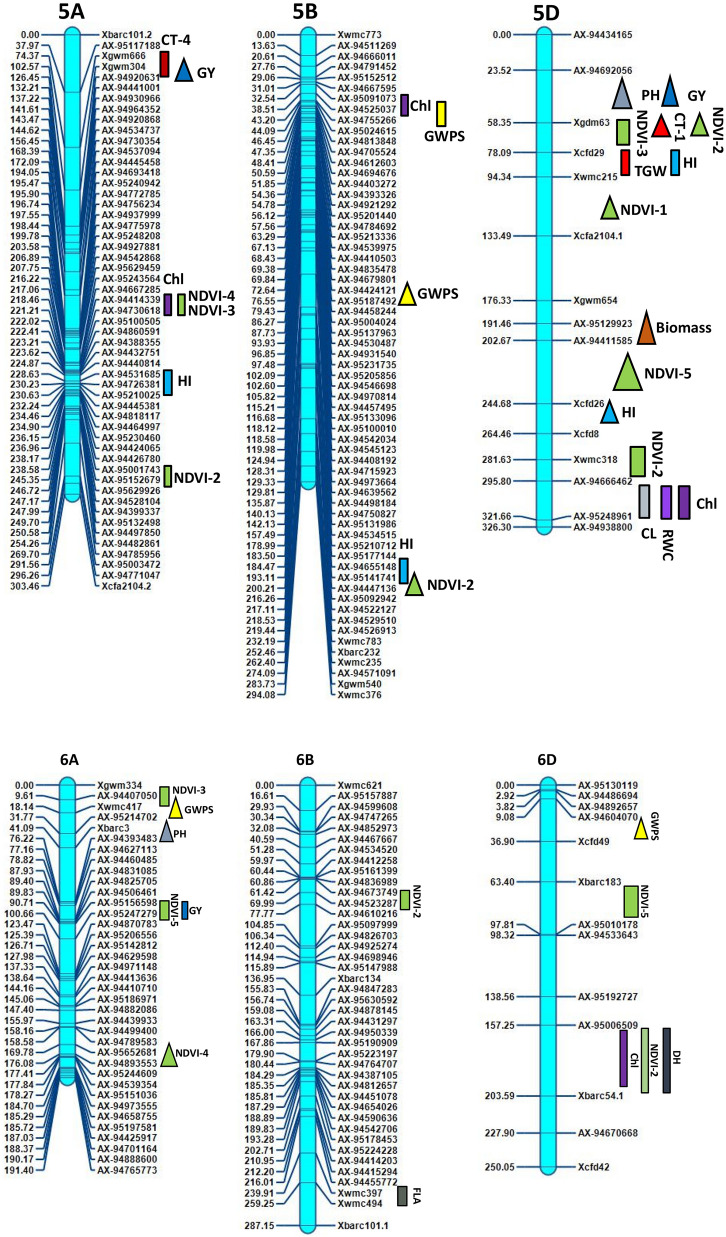

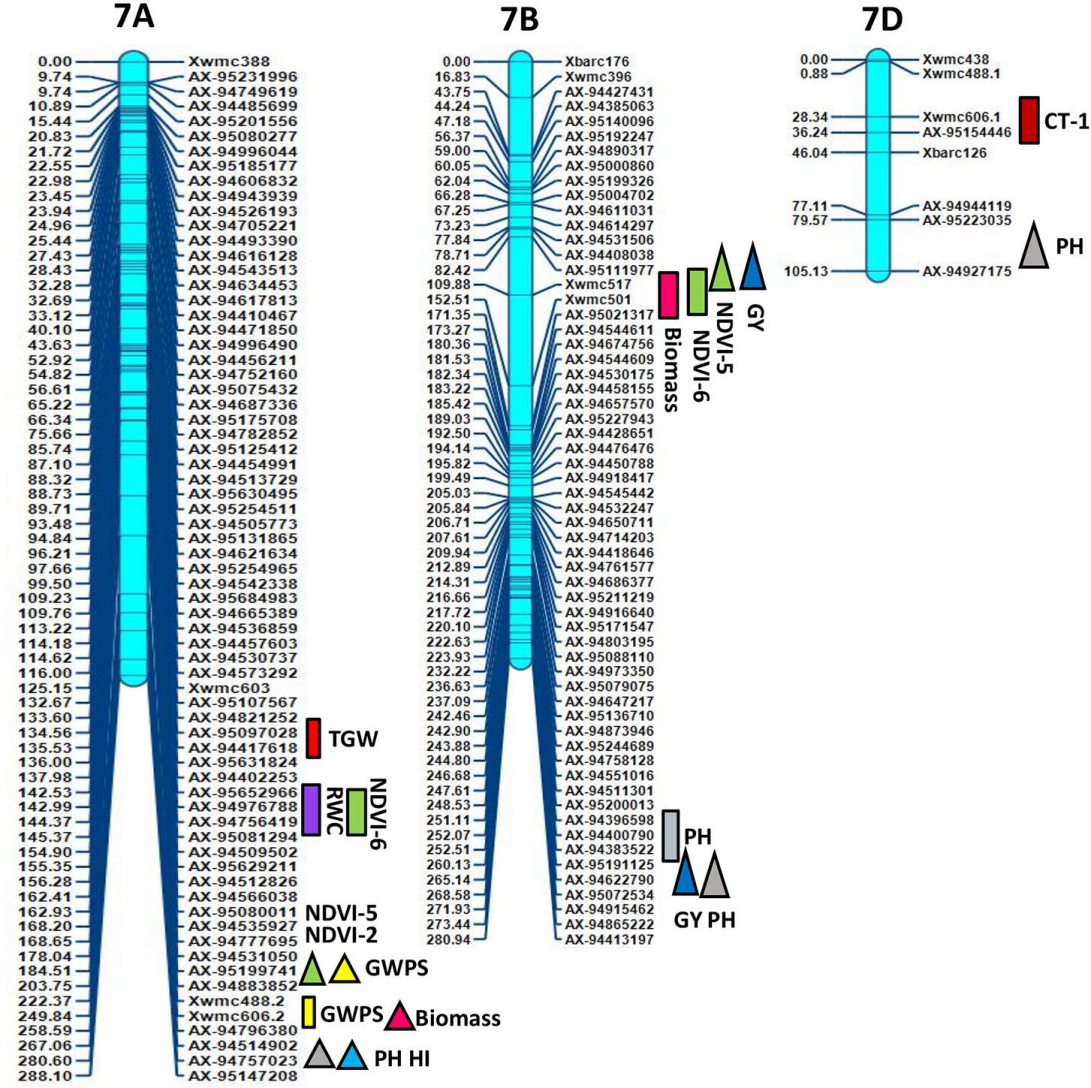
Genetic map of chromosomes showing QTL for physiological and yield component traits in the HD2733/2*C306 BILs population. Traits are projected as solid bars with different colours for which the legend is given at the end of figure. Rectangle locus represents main effect QTLs. Triangle locus represent epistatic effect QTLs. TGW, thousand grain weight, PH, plant height, GWPS, grain weight per spike, FLA, flag leaf area, CL, coleoptile length, AL, awn length, NDVI, normalized difference vegetative index, Chl, chlorophyll content, CT, canopy temperature, biomass, RWC, relate water content, DH, days to heading, HI, harvest index, GY, grain yield. IciMapping 4.1 software; http://www.isbreeding.net/software/default.aspx?type=detail&id=20 META-R, version 6.0: https://data.cimmyt.org/dataset.xhtml?persistentId=hdl:11529/10201.

Inclusive composite interval mapping (ICIM) investigation resulted in the detection of 43 genomic regions controlling 14 physiological and yield component traits based on individual location and year (Table 2 and Fig. 2). These genomic regions were found scattered over 18 chromosomes excluding 1B, 3A and 3D. Most QTLs were detected on chromosomes 2A, 5D, 5A and 4B, which had 9, 7, 5 and 4 QTLs, respectively (Fig. 2) with LOD values ranging from 2.51 to 11.24. The QTLs ranged from those responsible for phenotypic variance from 2.1 to 28.35%, involving nine major QTLs of which 6 were stable over more than one environment.

### QTLs analysis for physiological traits

ICIM analysis revealed thirty six putative QTLs for four physiological traits NDVI, CT, chlorophyll content, CL and RWC measured over seven environments (Table 2). The QTLs explained 2.10% to 28.35% phenotypic variance in different environments. Out of 36 QTLs expressed for variation in physiological traits, 18 belonged to A genome, 12 to D and 6 to B genome, but quite scattered in the genome. The common chromosomes housing QTLs functioning for moisture deficit stress tolerance were on chromosomes 2A, 5A, 5D, 6A and 6D (Table 2, Fig. 2).

**Figure 3:**
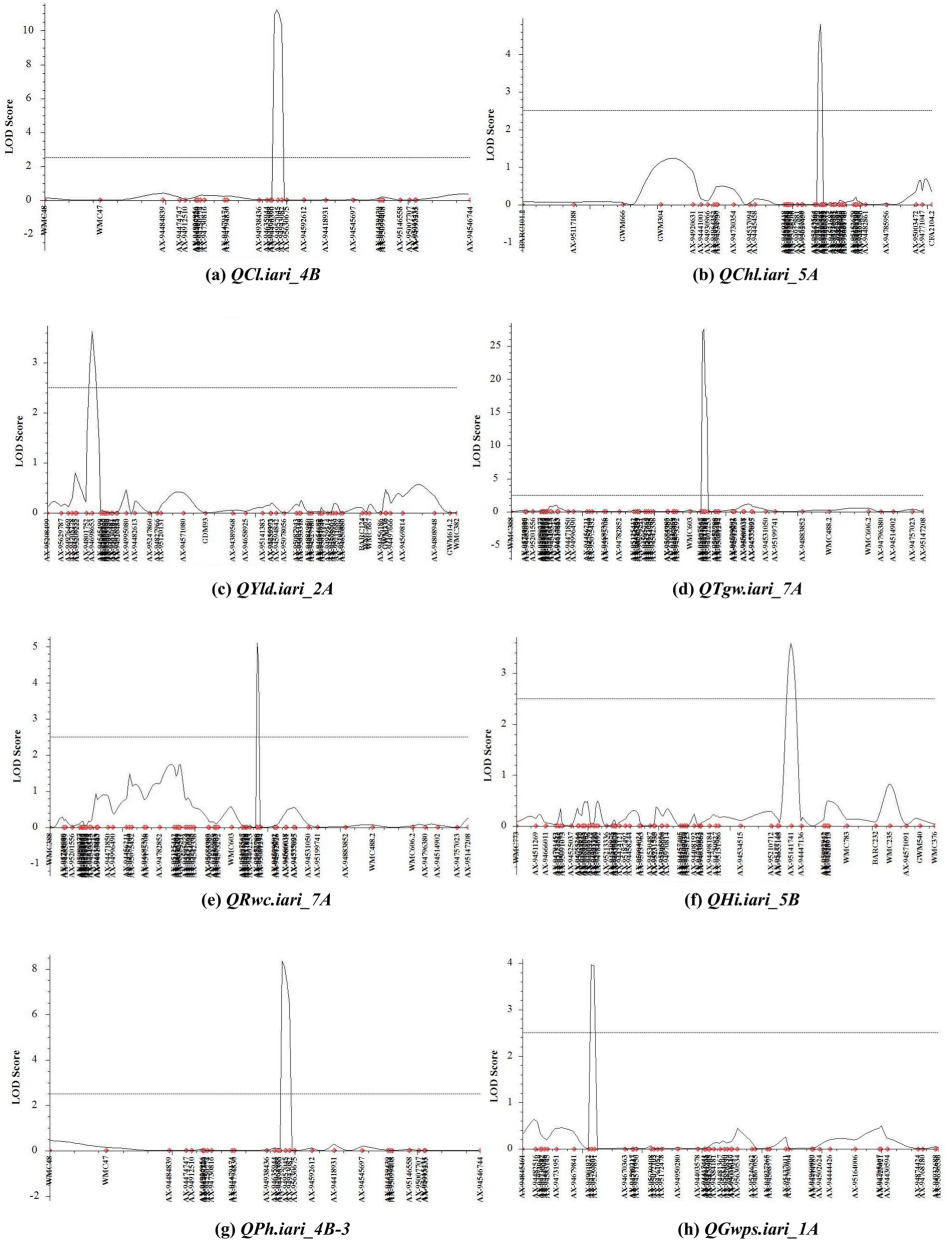
LOD threshold graphs of (**a**) coleoptile length (**b**) chlorophyll content (**c**) grain yield (**d**) thousand grain weight (**e**) relative water content (**f**) harvest index (**g**) plant height (**h**) grain weight per spike.

15 QTLs affecting NDVI measured at different intervals of crop growth period were located on nine different chromosomes 2A, 5A, 5D, 6B, 7A, 6D, 6A, 2D and 7B (Table 2). Ten QTLs represented NDVI expression variation at booting stage (NDVI-2) followed by milk stage of grain formation (NDVI-3). One pair of QTLs, *QNdvi1.iari_5D and QNdvi3.iari_5A* was simultaneously detected in two sites; E1; E3 and E1; E2 (Table 3). We identified 10 candidate genes (CGs) underlying QTL, *QNdvi3.iari_5A*, which also colocalized with chlorophyll content (Supplementary Table S6). The positive allele for QTL, *QNdvi1.iari_5D* contributed from HD2733 and *QNdvi3.iari_5A* was derived from C306. For NDVI-2, the maximum percent of phenotypic variance was explained by QTL *QNdvi2.iari_2A* on 2A chromosome. Seven QTLs of NDVI shared high confidence intervals with multiple traits viz HI, GY, biomass, RWC, DH, CT and SPAD. In total, 8 QTLs were identified for chlorophyll content and explained phenotypic variance from 2.10 to 9.30%. Ten digenic epistatic QTLs were identified for NDVI with a range of PVE from 4.89 to 14.61%. Two QTLs on chromosome 2A and 5D with a significant additive effect (0.025 and -0.023) interacted with locus on 3A and 7A respectively (Table 4). The QTL on 5A (*QChl.iari_5A*) and 6D (*QChl.iari_6D*) seemed to be important QTL for chlorophyll content, given the genomic regions found coinciding with NDVI (Figs. 2 and 3). Interestingly, HD2733 contributed positive alleles for most of the QTL falling under chlorophyll content. One main effect QTL on chromosome 3B (*QChl.iari_3B)* showed digenic interaction with locus on 4D (Table 4). On chromosome 7A and 5D, we found an association for RWC, with an average LOD score value of 3.81. Notably, QTL on 7A *QRwc.iari_7A*, carrying positive allele from C306, explained about 10.77% of phenotypic variance (Figs. 2 and 3). Additionally, the 5D region was found to be co-located with chlorophyll content QTL. On the other hand, five chromosomal regions were associated with canopy temperature dispersed over 2A, 5A and 7D. Coleoptile length genomic locations were positioned over chromosome 4B and 5D, indeed maximum phenotypic variance of 12.63% to 28.35% was explained by the QTL *QCl.iari_4B* and *QCl. iari_5D,* respectively (Fig. 3).

**Table 3:** List of stable QTLs consistently detected between different locations in BILs of HD2733/2*C306.

Trait	Location	QTLs	Position	Marker interval	LOD	PVE (%)	Additive effect	Confidence interval (cM)
DH	E6 E1	*QDh.iari_6D*	183	AX-95006509-Xbarc54.1	3.10	6.23–6.99	5.06	169.5–193.5
GWPS	E6 E5	*QGWPS.iari_5B*	42	AX-94525037-AX-94755266	3.70	7.71–9.03	− 0.94	39.5–44.5
PH	E7 E2	*QPh.iari_4B-2*	95	AX-94496390-AX-94957045	3.56	8.48–13.92	− 3.30	93.5–96.5
PH	E5 E1	*QPh.iari_4B-3*	104	AX-95630675-AX-94592612	5.57	9.31–13.66	− 3.74	99.5–110.5
NDVI-1	E2 E3	*QNdvi1.iari_5D*	282	Xwmc318-AX-94666462	3.65	5.71–7.40	0.074	274.5–288.5
NDVI-3	E2 E1	*QNdvi3.iari_5A*	221	AX-94414339-AX-94730618	2.67	4.58–8.08	− 0.016	218.5–222.5

E1-Delhi rainfed (2016–2017), E2-Delhi irrigated (2016–2017), E3-Indore restricted irrigation (2016–2017), E4-Powarkheda rainfed (2016–2017), E5-( Delhi rainfed (2017–2018), E6-Delhi irrigated (2017–2018) and E7-Indore restricted irrigation (2017–2018).

**Table 4:** QTL × QTL epistatic interactions identified for various physiological and yield component traits.

ENV	Trait	Ch-Ini	Position	Marker interval (i)	Ch-Inj	Position	Marker interval (j)	LOD	PVE (%)	AAij
E5	NDVI-1	2D	70	AX-95091154-AX-94983022	5D	110	Xwmc215-Xcfa2104.1	3.46	7.13	− 0.038
E2	NDVI-2	3D	0	AX-94543536-AX-94575413	5B	200	AX-95141741-AX-94447136	4.72	6.59	0.069
E2	NDVI-2	2A	250	AX-95633318-AX-94881950	2A	300	AX-94650880-Xbarc124	4.33	5.52	− 0.086
E3	NDVI-2	5D	70	Xgdm63-Xcfd29	7A	195	AX-95199741-AX-94883852	4.31	12.46	− 0.721
E6	NDVI-3	3B	75	AX-94462966-AX-94532649	3D	50	AX-94519760-Xgwm52	3.23	6.36	− 0.413
E3	NDVI-3	2A	340	AX-94497666- AX-94569814	3A	60	AX-94941121-AX-94998102	4.76	14.61	− 0.394
E1	NDVI-4	1B	100	AX-94507852-AX-94490037	4A	175	AX-94881522-Xgwm165	3.17	5.33	0.0311
E1	NDVI-4	1D	225	Xcfa2147-AX-94730813	6A	175	AX-95652681-AX-94893553	3.56	4.89	0.034
E6	NDVI-5	3B	50	AX-95630213-AX-95630742	5D	225	AX-94411585-Xcfd26	3.32	8.63	− 0.032
E6	NDVI-5	7A	200	AX-95199741-AX-94883852	7B	100	AX-95111977-Xwmc517	3.84	5.64	0.025
E6	CT-1	1D	125	AX-94466473-AX-95189966	5D	75	Xgdm63-Xcfd29	3.27	10.13	0.0695
E6	Chl. content	3B	210	Xwmc689-Xwmc78	4D	25	AX-95257633-Xcfd23	4.95	6.19	0.5099
E5	Chl. content	1B	40	Xwmc44- AX-94780679	4D	85	Xwmc825- AX-94864643	4.41	1.21	− 1.3624
E2	PH	2A	375	AX-94808948-Xgwm614.2	7D	100	AX-95223035-AX-94927175	4.30	5.63	− 4.266
E3	PH	6A	75	Xbarc3-AX-94393483	7A	275	AX-94514902-AX-94757023	5.78	10.47	5.0778
E3	PH	5D	30	AX-94692056-Xgdm63	7B	265	AX-95191125-AX-94622790	4.10	7.96	− 4.6201
E3	GWPS	1D	225	Xcfa2147-AX-94730813	6D	30	AX-94604070-Xcfd49	4.33	5.87	1.1403
E6	GWPS	3A	225	Xwmc627-Xbarc54.2	5B	75	AX-94424121-AX-95187492	3.05	6.24	0.4483
E6	GWPS	6A	25	Xwmc417-AX-95214702	7A	200	AX-95199741-AX-94883852	3.11	5.77	− 0.3858
E4	TGW	2B	175	AX-95142963-AX-95112013	3D	50	AX-94519760-Xgwm52	4.05	8.61	− 0.8412
E6	biomass	5D	200	AX-95129923-AX-94411585	7A	250	Xwmc606.2-AX-94796380	3.57	7.03	− 17.716
E1	GY	3B	75	AX-94462966-AX-94532649	7B	100	AX-95111977-Xwmc517	3.03	7.01	− 24.4711
E3	GY	2B	155	AX-94493467-AX-94703320	5A	125	Xgwm304-AX-94920631	4.17	11.26	− 26.538
E3	GY	5D	25	AX-94692056-Xgdm63	7B	265	AX-95191125-AX-94622790	5.38	4.01	− 25.244
E1	HI	4A	75	AX-94543483-AX-95096811	4D	25	AX-95257633-Xcfd23	3.24	6.28	− 0.0183
E2	HI	5D	250	Xcfd26-Xcfd8	7A	275	AX-94514902-AX-94757023	4.06	5.79	0.0467

1) AAij, epistatic effects), Position of QTL located on chromosome: as cM distance from the top of each map.

LOD, A LOD threshold of 3 was used for declaration of QTL,

PVE (%), Phenotypic variance explained by QTL.

Location code; E1-Delhi rainfed (2016–17), E2-Delhi irrigated (2016–2017), E3-Indore restricted irrigation (2016–2017), E4-Powarkheda rainfed (2016–2017), E5-Delhi rainfed (2017–2018), E6-Delhi irrigated (2017–2018) and E7-Indore restricted irrigation (2017–2018).

### QTLs analysis for yield component traits

Altogether 27 QTLs were identified as significantly linked with yield component traits, residing over 1A, 2A, 4A, 6A, 7A, 2B, 4B, 5B, 6B, 7B, 2D, 5D and 6D chromosomes, using seven environment data (Table 2 and Fig. 2). Among these, *QPh.iari_4B-2* and *QPh.iari_4B-3* for PH consistently associated with trait increase in two environments. Here, *AX-94496390* is the nearest marker for QPh.iari*_4B-*2 and *AX-94592612* for *QPh.iari_4B-3* with 11.35% of average explained phenotypic variance (Figs. 2 and 3). Particularly, *QPh.iari_4B-3* region carries 11 candidate genes. Meanwhile, putative QTL, *QPh.iari_7B* flanked by markers AX-94622790 and AX-95072534 was associated with trait decrease, deriving positive allele for reduced PH from semi-dwarf parent, HD2733.

In particular, one locus *AX-95151743-AX-94877462* at chromosome 2D showed QTL for important yield component trait flag leaf area, with 2.91 cm^2^ of additive effect and high PVE (11.44%). Along with this, we observed QTL for AL on chromosome 4A *QAl.iari_4A*. There were about four QTL identified having alleles for TGW, spread over 2A, 2B, 7A and 5D chromosomes (Figs. 2 and 3). Among these, the region between markers *AX-94995080-AX-94482613* on chromosome 2A, harbored major QTL *QTgw.iari_2A*, showing 11.20% of the phenotypic variance. This locus derived positive allele from HD2733, with an additive effect of 3.2 g for trait increase and found to be associated with 6 candidate genes (Supplementary Table S6). GWPS is also a key indicator for yield increase in wheat and, we found a stable QTL locus *QGwps.iari_5B,* between rainfed and irrigated condition, showing an average of 8.37% phenotypic variance (Table 3). Other QTLs for GWPS were located over 1A and 7A chromosomes (Figs. 2 and 3).

In addition to the above yield component traits, the presence of 5 significant QTLs was detected for HI and 3 for GY, with PVE ranging from 6.00 to 8.31% (Figs. 2 and 3). Among these, three QTLs of HI*,* derived positive allele from HD2733, *QHi.iari_2D*, *QHi.iari_5D*, *QHi.iari_5A* were found co-located with loci for NDVI, TGW and chlorophyll content, respectively. Here, allele contributing for NDVI derived from C306 and for TGW, chlorophyll content was from HD2733. Whereas, QTL for GY on chromosome 6A *QYld.iari_6A* was found to be associated with NDVI. On chromosome 6D, there was a stable QTL *QDh.iari_6D* located for DH under both E1 and E6 environment, explaining 6.61% average PV. Two QTLs of GY, *QYld.iari_2A* and *QYld.iari_2B* combinedly associated with 11 CGs. Also, three epistatic QTLs were detected for GY, a QTL showing both main effect and interactive effect found on chromosome 2B, *QYld.iari_2B*. Particularly, this locus interacted with a main effect QTL for CT *QCt4.iari_5A*, explaining phenotypic variance of 11.26% for GY (Table 4). One QTL, *QBiomass.iari_7B* has been identified for biomass under E2 condition depicting PV of 10.32%. This also co-localized with the QTL for NDVI, positioned between markers *Xwmc517-Xwmc501* (Table 2).

On examining the presence of candidate genes in the QTL confidence regions,78 genes were observed in 11 genomic regions (Supplementary Table S6). The candidate genes were present in the physical location of the QTLs identified for TGW, GY, HI, GWPS, NDVI, chlorophyll content, RWC and, PH. These candidate genes belonged to protein kinases (fructokinase, serine/ threonine protein kinase, phosphoglycerate kinase), transporters (magnesium, calcium), signaling molecules (SCP, auxin response factor) and, transcription factors (Homeodomain leucine zipper) among many others.

## Discussion

Both physiological and yield component traits dispersed among diverse parental lines need to be combined cumulatively to design the recombinant genotype that makes the water use efficient variety, better than the parental genotypes. The breeding procedure for introgression of genomic regions housing alleles that facilitate adaptation against drought and heat into such genotypes improves the performance of the selected genotype under restricted irrigation and rainfed ecosystems^10,24^. The choice of the traits that contribute to the adaptation to abiotic stress is the first step. Earlier studies have successfully established the association and usefulness of NDVI, chlorophyll content, CL and CT as a selection tool under moisture stress condition for improving grain yield in wheat^6,14,25–27^. Therefore, for proper exploitation of these traits, there is a need to decipher their mechanism in diverse genotypes in the context of moisture stress tolerance. Highly stable with introgressed favorable moisture deficit stress tolerance traits in backcross inbred lines were identified by Manu^28^. The donor genotype C306 is one of the most well-adapted variety for rainfed conditions across different wheat growing ecosystems and is identified as a good combiner parent to transfer moisture deficit stress tolerance in spring wheats. The backcrossed inbred lines (BC_1_F_6_) of HD2733/2*C306 were screened and selections practiced after phenotyping for 2 years for physiological and yield component traits under unirrigated conditions in the north-western plains, the largest wheat producer region in south-east Asia followed by the central Indian region, known for best grain quality wheat to identify the best recombinant inbreds to tackle the depleting water table causing restricted irrigated agronomy. The BIL populations were exposed to moisture stress during the entire growing season over the locations under rainfed situation that also included situations of flash flooding in the same season as total drought at different growth stages, enabled recording of the adaptation potential of the selections, justifying the attempt to map the genomic regions that may harbor QTLs for physiological traits expressing under moisture deficit or abundance stress.

The presence of a significant difference between parents as well as wide range of variations for NDVI, CT, RWC, chlorophyll content, CL, TGW, GWPS and GY traits in BILs, indicated the existence of allelic variations for each trait. High heritability of traits and positive phenotypic correlation between NDVI, SPAD, TGW, GWPS, biomass with GY indicate the importance of traits in breeding varieties for moisture deficit stress tolerance. CT estimates from post-flowering stage are negatively correlated with GY under moisture stress, hence cooler canopy associated with higher GY^29^. The genotypes within an inbred BC_1_F_6_ population segregated in an expected 3:1 ratio, that could be visualized by data from graphical genotyping. We constructed a genetic linkage map of the HD2733/2*C306 covering 4700.15 cM using 35K Axiom genotyping array integrated with SSR markers, that closely agrees with previously reported maps with genome coverage ranging from 1070 to 4223.1 cM^30^. Low polymorphism in wheat, is a limiting factor to develop high density linkage map, the presence of homeologus chromosomes and a large proportion of repetitive sequences reduces the chance of getting polymorphic markers across the genome in wheat^17,31^. We observed dissimilarity in markers coverage, having 87.5% of markers placed over B and A genome, remaining 12.5% on D genome. SNP genotypic platform Axiom 35K Breeders Array has a biased distribution of SNP probes, having few markers mapped to the D genome. This may have resulted in the low polymorphism observed on the D genome. Somers^32^ and Wang^33^ also reported uneven marker coverage due to low diversity in D genome.

NDVI is an integrative measure to estimate chlorophyll, early vigour and plant biomass. This trait can be modeled for capturing dynamics of photosynthesis decay under moisture stress^18^. QTLs for NDVI during grain filling stage (NDVI-4 to NDVI-6) explained about 45.6% cumulative phenotypic variance. These QTLs could be exploited for the stay-green trait. The QTL *Qndvi6.iari_2A* located on chromosome 2A linked to the marker *Xgdm93* influences variation in NDVI as earlier identified by Olivares-Villegas^34^. *QNdvi3.iari_6A* within the marker interval AX-94407050-Xwmc417 was also associated with the marker *Xwmc417* in a previous study with RILs generated from the cross DBW43/HI1500 in this lab^35^ and found to be linked with MQTL 49 which host QTLs for photosynthesis and TGW under drought as well as heat condition^36^. Hence, *Xwmc417* is an important marker associated with NDVI under moisture stress condition. *QNdvi2.iari_6D* was co-located with *QChl.iari_6D* and *QDh.iari_6D* flanked between *AX-95006509-Xbarc54.1*, where QTL for leaf senescence rate *QLSR.caas-6D* was identified between *Xbarc54- Xbarc365* by Li^37^. Therefore, this QTL is important for improving chlorophyll content and photosynthetic efficiency. Overall, including those reported earlier^7,16,38,39^ 15 QTLs were found associated with NDVI, spread over chromosomes 2A, 2D, 5A, 5D, 6A, 6D, 6B, 7A, and 7B. Co-localization of detected QTL of NDVI with other physiological traits such as chlorophyll content, RWC and yield components GY, HI, biomass was observed. The occurrence of colocalization of QTL for NDVI with TGW, GY, SL, PH, and kernel number spike was reported by Gao^16^. Thus, in our study NDVI is significantly correlated with GY, suggesting the integrity and effectiveness of NDVI QTLs in screening for tolerance to moisture stress conditions.

The role of chlorophyll content of flag leaf influencing grain yield is documented as a criterion for selection for drought and heat tolerant wheat^40^. The trait was targeted for identifying the BILs with moisture deficit stress tolerance potential in this study. Among the observed QTLs for chlorophyll content on chromosomes 2A, 4A, 5A, 3B, 5B, 5D and 6D, some were also reported as mapped to similar locations in earlier studies^16,26,39,41,42^. Of these, an average phenotypic variance of 8.35% was explained by QTLs on chromosome 5A, 3B and 5D, previously these genomic regions were noted for chlorophyll content by Yang^43^ and Zhang^44^ under different water stress environments. Among the two epistatic QTL identified, the locus on chromosome 1B linked with *Xwmc44*; earlier this SSR marker linked with reported QTL for flag leaf staygreeness (*Qspad.acs-1B.4*)^43^*.*

Prominent expression of phosphoglycerate kinase is known to be associated with improved saline tolerance due to higher chlorophyll retention and enhanced proline accumulation^45^, hence the presence of this CG in the QTL region of *QChl.iari_2A* hints this as an important QTL to improve chlorophyll content under moisture stress. Genomic locations of QTLs for canopy temperature coincides with the results obtained by Diab^46^, Mason^47^ and Pinto^27^. The flanked region on 7D between *Xwmc606.1-AX-95154446* positioned QTL for CT, *QCt1.iari_7D* with PVE of 9.26%. The SSR marker *Xwmc606* was found to be linked with MQTL 64, which includes QTLs for photosynthesis and water-soluble carbohydrates under drought stress. Our donor parent contributed the positive allele for novel QTL *QRwc.iari_7A* mapped on chromosome 7A that explained PV of 10.77%. There are no earlier reports of QTLs detected for RWC on 7A chromosome while we could locate a novel QTL *QRwc.iari_7A* located on 7A, in the region *AX-94976788-AX-94756419*.

For the yield component traits examined in this study, one major QTL for TGW was detected on chromosome 2A corresponding to the location reported in earlier studies under drought stress^11,12,48^. Ain^49^ and sukumaran^9^ through genome-wide association studies identified marker trait association and Guan^50^ reported QTL cluster for TGW on chromosome 2A, hence this can be an important region to be targeted for improving TGW. The region *AX-95097028-AX-94417618* on chromosome 7A contained a QTL for TGW (*QTgw.iari_7A*). Gahlaut^4^ and Wang^51^ reported a major QTL for TGW on chromosome 7A in the corresponding position, which may be the extended region of the same QTL differing from those occurring in the donor parent C306 used here. Previous investigations highlighted co-location of QTL for reduced CL with GA-insensitive *Rht-B1b* and *Rht-D1b* dwarfing genes^14,52^. The presence of dwarfing genes in semi-dwarf varieties reduces CL and early vigour, an avoidable situation when selecting for moisture deficit stress tolerance in wheat. Long CL (9.94 cm) of C306 cultivar, increased ability to emerge from deep sowing for rapid establishment with higher leaf growth and early ground cover under moisture stress soils^53^. Two QTLs *QCl.iari_4B* and *QCl. iari_5D* were detected for long CL explaining a variation of 28.35 and 12.64%, respectively. The co-localization of the QTL *QCl.iari_4B* with QTL (*QPh.iari_4B-2*) for PH indicated their physiological adaptation as a QTL complex in wheat adapted to moisture deficit stress tolerance. This is in agreement with previous reports where Rebetzke^14^ mapped major QTL for the *Rht-B1* locus on chromosome arm 4BS associated with CL explaining 27–45% of the genotypic variance. Besides, Li^54^ mapped SNP (IWA8564) linked to coleoptile length QTL, *QCL.stars-4BS* on chromosome 4B while other studies have also reported some QTLs mapped to regions on chromosomes 1A, 2B, 2D, 4A, 5A, 5B, 5D, 6A and 6B for CL^14,52,55^; *Lcol-A1* being the prominent one on chromosome 1A^55^. We could locate another QTL *QCl. iari_5D* with a similar influence on CL on chromosome 5D. The QTLs for CL on chromosomes 5D and 4B along with two stable QTLs for PH on chromosome 4B should enable desired plant height selection with long coleoptile as these QTLs have been known to show an average phenotypic variance of more than 10%^50,56^. However, in such criteria, it was also important to avoid the QTLs like *QPh.iari_7B* which carries an allele for trait decrease having originated from dwarf parent HD2733, whose region coincides with that reported earlier^7,15^. Further, the lipoxygenase gene found in the region of *QPh.iari_4B-3*, is reported to be associated with photosynthetically active radiation and quantum yield water index on chromosome 4B under rainfed situation^57^.

Masoudi^58^ identified a QTL for awn length on chromosome 4A flanked by marker interval *wPt-5730-tPt-5597*. Our study detected a QTL *QAl.iari_4A* near to the same locus for awn length flanked between *Xbarc78-AX-94694992*. Previously, Acuña‐Galindo^36^ identified *Xbarc78* as a linked marker to MQTL32, which is associated with drought and heat tolerance. Genomic regions for FLA were reported on 2D^59,60^ and 6B^61^. Interestingly, Wu^60^ reported that genomic region on chromosome 2D harbor QTL cluster for FLA, flag leaf width and flag leaf length. hence, the major QTL, *QFla.iari_2D*, positioned between *AX-95151743-AX-94877462* could be beneficial in breeding for increasing FLA under moisture stress. . In our study, *Xwmc397* is linked with FLA on chromosome 6B. QTLs identified for GY on 2A may be similar to those reported earlier^13,48,51^. The locus on chromosome 6A (*AX-95247279-AX-94870783*) was associated with GY and NDVI-5, earlier a QTL on chromosome 6A linked to GY, TGW and green canopy duration was reported by Sukumaran^9^ and Simmonds^62^. Though QTLs for DH in wheat are associated with photoperiodism (Ppd-A1, Ppd-B1, Ppd-D1) and vernalization (VRN-A1, VRN-B1, and VRN-D1) along with some others, a novel, but consistent QTL for DH was detected here on chromosome 6D (*QDh.iari_6D*) which is likely to be not associated genome location-wise with either Ppd or VRN loci. Though QTL for biomass has been located on 7B earlier^7^, we could locate a major QTL (*QBiomass.iari_7B*) on chromosome 7B (110.5–136.5 cM), which was also found to be associated with NDVI in the irrigated environment making the pair useful for selection of wheat adapted to rainfed ecosystem. A stable QTL for GWPS (*QGwps.iari_5B*) was identified in the interval *AX-94525037-AX-94755266* of chromosome 5B that seemed to be different from another QTL reported^63^ on chromosome 5B. While another QTL for GWPS on 7A between *Xwmc488.2-Xwmc606.2* were co-localized with QTLs associated with grain yield traits HI and GY^64^. Likewise, on chromosome 7A in the Nongda3338/Jingdong6 doubled haploid (DH) population, a QTL rich region associated with GWS, GNS and TGW was reported by Guan^50^. Out of 5 QTLs detected for HI, three QTLs located on 2D, 5D and 5A were pleiotropic in nature coinciding with NDVI, TGW and chlorophyll content, respectively. Kumar^65^ and Schmidt^66^ detected colocalized QTL for HI on chromosome 2D with yield component traits. In this study, 26 digenic epistatic QTLs were identified for physiological and yield component traits in the seven environments; this indicates a complex genetic mechanism involved in moisture stress tolerance in wheat^67^. Many of the locus which failed detection in phenotype but their interaction with other locus may show a significant effect for the trait under consideration. Among 26 QTLs, four QTLs were main effect, earlier studies of Gahlaut^4^ and Shukla^15^ reported QTL × QTL interactions for yield component traits in wheat.

Overall, it was possible to identify QTLs or QTL clusters co-localized on chromosomes 2A, 5A, 6A, 7A, 4B, 7B, 2D, 5D, and 6D with more than two traits. Early studies have reported co-localization of QTLs for yield traits^16,68^. The interval between 94.5 and 98.5 cM on chromosome 4B is a significant locus carrying QTLs for longer CL and PH. These QTL clusters indicate the pleiotropic nature or linkage effect of genes residing in their genomic regions. Additionally, a region between 218.5 and 230.5 cM on chromosome 5A impacted with QTLs associated with NDVI, chlorophyll content and HI. This result was supported by Gahlaut^4^ who reported specific genomic regions on chromosome 5A associated with yield component traits.

Candidate drought-responsive genes exhibit various patterns in sensing and transduction of signal, scavenging reactive oxygen species, osmotic regulation, carbohydrate and, fatty acid metabolism. Identification of such candidate genes in QTL genomic regions will pave the way for precision breeding in the future. Plants have evolved comprehensive mechanisms to adapt to moisture stress. Among these, cuticular wax synthesis is one such mechanism that provides an essential barrier to protect plants from water loss. The enzymes involved in acyl reduction pathway plays a major role in cuticular wax deposition^69^. Our study observed several genes (fatty acyl CoA reductase, Keto-acyl synthase, acyl carrier protein) in the QTL regions that were involved in fatty acid synthesis and reduction pathways as well as glycolytic pathways (phosphofructokinases) that are important for the resistance response of plants to moisture stress. In this study, candidate genes observed in certain genomic locations belong to protein degradation pathways such as ubiquitin-conjugating enzymes, proteases essential for removing proteins that have denatured due to stress. Furthermore, proteins involved in sensing, and signaling stress response which are key regulators of downstream genes in the response pathway were present in QTL regions. These included protein phosphatase 2C, auxin response protein, cation/proton exchangers and, calcium transporting ATPase etc. Arabidopsis protein phosphatase 2C has an established role in abscisic acid (ABA) signaling, an important event in moisture stress response^70^. Calcium transporting ATPase is also known to be a positive regulator of drought tolerance and an increase in the cytoplasmic Ca^2+^ concentration is an important step in early ABA signaling^71^. Thus, various candidate genes identified at 11 genomic locations in this study might be controlling the underlying phenotypic traits and future gene expression studies might unravel their role in moisture stress tolerance.

## Conclusion

QTL mapping using BILs population identified many genomic regions associated with physiological and grain yield component traits under moisture deficit stress tolerance. This includes nine putative major QTLs, 26 digenic epistatic QTLs and six stable QTLs on 4B, 5A, 5B, 5D, 6D for PH, NDVI, GWPS and DH, respectively. In total, 43 genomic regions were detected for 14 traits across seven independent experiments. Our results will facilitate exploiting identified genomic regions for using in MAS and MARS approaches for the development of moisture deficit stress tolerant genotypes.

## Materials and methods

### Plant materials and field trials

The mapping population consisting of 183 BC_1_F_6_ lines derived from a cross between HD2733/2*C306. HD2733 is a superior, high yielding, Indian wheat variety released from IARI, New Delhi for timely sown, irrigated condition. The BILs population along with their parents were grown in three locations representing two wheat growing zones in India, under different moisture regimes viz* Rainfed* (E1, E4, E5), *Restricted irrigation* (E3, E7) and *irrigated* (E2, E6) condition over two seasons (2016–2017 to 2017–2018). The population was screened under both rainfed as well as irrigated condition at Delhi (E1, E2, E5, E6) and only under restricted irrigated condition at Indore (E3, E7) for two seasons. At Powarkheda (E4), the BILs were evaluated for one season under rainfed condition only, thus making a total of seven independent experiments under the study (Table 1). The trials were raised in an alfa lattice design with two replications. Each genotype was grown in a plot of 3 rows of two-meter length, keeping 30 cm row to row distance and 10 cm plant to plant distance. The recommended wheat cropping practices were adapted for proper crop establishment at all locations. The details of the three locations are presented in Table 1. For rainfed condition only one irrigation was given at 21 days after sowing (DAS) and for restricted irrigation two irrigation were given (21 DAS and 40 DAS). Irrigated environment was provided with five irrigations for the establishment of crop. The soil moisture content (w/w) across locations was estimated by gravimetric method at depth of 15 cm in the root zone and their details are represented in Fig. 1.

### Phenotyping

The data on fifteen physiological and yield components traits were recorded according to the standard procedure detailed in the field guide of CIMMYT^72^. These included: repetitive measure of NDVI, CT using Green seeker and infrared thermal gun at different intervals of crop growth period denoted as NDVI-1:before heading, NDVI-2:booting stage, NDVI-3:milky stage, NDVI-4:late milky stage, NDVI-5:dough stage, NDVI-6:ripening stage and for CT represented as CT-1:vegetative stage, CT-2:early milk stage, CT-3:late milk stage and CT-4: ripening stage, respectively. The chlorophyll content of flag leaf was scored using SPAD-502 Minolta, chlorophyll meter and randomly selected 5 plants fresh leaf samples were assessed to get RWC. Following these traits BILs were also phenotyped for CL, days to heading (DH), awn length (AL), flag leaf area (FLA), PH, GWPS, TGW, biomass, harvest index (HI) and GY.

### Phenotypic data analysis

Anova, Mean, standard deviation, range, heritability and phenotypic correlation analysis for each trait were calculated using PROC GLM in SAS. 9.3 software^73^. Phenotypic data were used to estimate the best linear unbiased prediction values (BLUP) of two replications of a given environment using META-R (Multi Environment Trial Analysis with R for Windows) software version 6.0^74^.

### Genetic linkage map construction and QTL mapping

Genomic DNA was extracted using CTAB method and DNA was quantified with nano drop and quality was determined using 1% agarose gel electrophoresis with λ DNA as the standard. The 35K Axiom genotyping array and SSR markers were used for genotyping of BILs population and the parents. Primer sequences of SSR markers for the Beltsville Agricultural Research Center (BARC), Gatersleben wheat microsatellite (GWM), Wheat Microsatellite Consortium (WMC), INRA Clermont-Ferrand (CFA, CFD) and Gatersleben D-genome microsatellite (GDM) were obtained from the Grain Genes website (http://wheat.pw.usda.gov/GG2/index.shtml) and synthesized.

The linkage map of BILs population was constructed with SNP and SSR markers using IciMapping 4.1 software. Map distances between markers were calculated with the Kosambi mapping function. QTL analysis was performed using inclusive composite interval mapping (ICIM) with IciMapping 4.1 software^75^ (http://www.isbreeding.net). BLUP Phenotypic values of each environment and genotypic data were used for QTL detection. Missing phenotypic data were deleted using the “Deletion” command. The walking speed chosen for all QTLwas1.0 cM, with *P* = 0.001in step wise regression. The LOD threshold of 2.5 with 1000 permutations was chosen for declaration of putative QTL. QTLs were named using Q + trait name abbreviation + research department + chromosome^76^. The candidate genes (CGs) which are localized within a QTL region were identified based on the positions of markers flanking the CI of the QTL and search for genes within physical intervals using wheat reference genomic sequence available in Ensemble Plants database (https://plants.ensembl.org/index.html).

## Supplementary Information

Supplementary Information.

## Data Availability

The datasets generated during and/or analysed during the current study are available at Division of Genetics ICAR-IARI from the corresponding author, Harikrishna on reasonable request.
