# Supplementary material for: Mapping genomic regions of moisture deficit stress tolerance using backcross inbred lines in wheat (Triticum aestivum L.)
Source: Sci Rep. 2020 Dec 10;10:21646. doi: 10.1038/s41598-020-78671-x (PMC7729395; doi:10.1038/s41598-020-78671-x)
Supplement: Supplementary file 1 — Supplementary Information. [file 41598_2020_78671_MOESM1_ESM.docx]

**Supplementary tables and figures**

**“Mapping genomic regions of moisture deficit stress tolerance using backcross inbred lines in wheat (*Triticum aestivum*. L)”**

Shashikumara Puttamadanayaka^4^, Harikrishna^1^*, Manu Balaramaiah^1^, Sunil Biradar^1^, Sunilkumar. V. Parmeshwarappa^1^, Niveditha Sinha^1^, Sai Prasad S V^1^, Mishra P. C^5^, Neelu Jain^1^, Pradeep K Singh^1^, Gyanendra P Singh^2^ & Kumble V Prabhu^3^*

^1^ICAR-Indian Agricultural Research Institute, New Delhi 110 012, India

^2^ ICAR-Indian wheat and Barley Research Institute, Karnal

^3^PPV & FR Authority NAAS complex, New Delhi 110012, India

^4^ICAR-Indian Grassland and Fodder Research Institute, Jhansi, 284003

^5^Jawaharlal Nehru Krishi Vishwa Vidyalaya, Jabalpur, Madhya Pradesh 482 004

**Supplementary table S1. Phenotypic performance and distribution parameters for physiological traits of parents and BILs in seven environments.**

| **Trait** | **Env** | **HD2733** | **C306** | **BIL min** | **BIL max** | **BIL avg** | **SD** | **H^2^** |
| --- | --- | --- | --- | --- | --- | --- | --- | --- |
| **SPAD** | **E1** | **45.26** | **50.36** | **37.1** | **54.8** | **45.96** | **2.57** | **0.62** |
|  | **E2** | **55.26** | **53.25** | **36.4** | **50.9** | **43.3** | **3.03** | **0.63** |
|  | **E3** | **47.52** | **49.36** | **25.9** | **63.4** | **49.24** | **3.52** | **0.51** |
|  | **E4** | **-** | **-** | **-** | **-** | **-** | **-** | **-** |
|  | **E5** | **45.25** | **51.36** | **35.35** | **51.12** | **45.23** | **2.57** | **0.56** |
|  | **E6** | **52.44** | **49.48** | **36.4** | **54.9** | **43.3** | **3.21** | **0.52** |
|  | **E7** | **48.10** | **50.10** | **37.34** | **55.14** | **47.75** | **3.18** | **0.72** |
| **NDVI1** | **E1** | **0.71** | **0.72** | **0.67** | **0.78** | **0.73** | **0.026** | **0.82** |
|  | **E2** | **0.76** | **0.74** | **0.77** | **0.80** | **0.78** | **0.027** | **0.43** |
|  | **E3** | **0.76** | **0.78** | **0.71** | **0.83** | **0.75** | **0.05** | **0.44** |
|  | **E4** | **0.72** | **0.75** | **0.68** | **0.79** | **0.71** | **0.08** | **0.40** |
|  | **E5** | **0.71** | **0.74** | **0.69** | **0.77** | **0.74** | **0.038** | **0.53** |
|  | **E6** | **0.72** | **0.70** | **0.72** | **0.79** | **0.74** | **0.03** | **0.45** |
|  | **E7** | **0.73** | **0.74** | **0.68** | **0.79** | **0.74** | **0.056** | **0.68** |
| **NDVI2** | **E1** | **0.63** | **0.67** | **0.52** | **0.77** | **0.64** | **0.09** | **0.85** |
|  | **E2** | **0.72** | **0.72** | **0.69** | **0.79** | **0.73** | **0.053** | **0.69** |
|  | **E3** | **0.62** | **0.65** | **0.64** | **0.72** | **0.62** | **0.058** | **0.52** |
|  | **E4** | **0.64** | **0.68** | **0.63** | **0.71** | **0.65** | **0.11** | **0.44** |
|  | **E5** | **0.65** | **0.65** | **0.65** | **0.74** | **0.70** | **0.047** | **0.62** |
|  | **E6** | **0.68** | **0.67** | **0.68** | **0.75** | **0.71** | **0.046** | **0.63** |
|  | **E7** | **0.65** | **0.67** | **0.61** | **0.76** | **0.65** | **0.09** | **0.45** |
| **NDVI3** | **E1** | **0.63** | **0.67** | **0.37** | **0.71** | **0.55** | **0.061** | **0.91** |
|  | **E2** | **0.66** | **0.63** | **0.65** | **0.72** | **0.69** | **0.069** | **0.81** |
|  | **E3** | **0.56** | **0.59** | **0.47** | **0.62** | **0.51** | **0.075** | **0.70** |
|  | **E4** | **0.44** | **0.49** | **0.55** | **0.62** | **0.57** | **0.064** | **0.67** |
|  | **E5** | **0.58** | **0.61** | **0.54** | **0.68** | **0.61** | **0.071** | **0.56** |
|  | **E6** | **0.58** | **0.53** | **0.58** | **0.65** | **0.61** | **0.047** | **0.67** |
|  | **E7** | **0.53** | **0.58** | **0.55** | **0.63** | **0.58** | **0.107** | **0.63** |
| **NDVI4** | **E1** | **0.57** | **0.59** | **0.23** | **0.68** | **0.51** | **0.07** | **0.92** |
|  | **E2** | **0.61** | **0.55** | **0.61** | **0.68** | **0.64** | **0.10** | **0.58** |
|  | **E3** | **0.45** | **0.49** | **0.39** | **0.58** | **0.47** | **0.102** | **0.41** |
|  | **E4** | **0.44** | **0.48** | **0.41** | **0.53** | **0.44** | **0.07** | **0.62** |
|  | **E5** | **0.47** | **0.51** | **0.42** | **0.63** | **0.52** | **0.08** | **0.47** |
|  | **E6** | **0.52** | **0.48** | **0.51** | **0.62** | **0.55** | **0.052** | **0.64** |
|  | **E7** | **0.48** | **0.52** | **0.43** | **0.51** | **0.49** | **0.08** | **0.56** |
| **NDVI5** | **E1** | **0.45** | **0.49** | **0.12** | **0.64** | **0.44** | **0.12** | **0.84** |
|  | **E2** | **0.43** | **0.45** | **0.43** | **0.51** | **0.46** | **0.14** | **0.77** |
|  | **E3** | **-** | **-** | **-** | **-** | **-** | **-** | **-** |
|  | **E4** | **-** | **-** | **-** | **-** | **-** | **-** | **-** |
|  | **E5** | **0.38** | **0.43** | **0.32** | **0.55** | **0.39** | **0.09** | **0.58** |
|  | **E6** | **0.45** | **0.39** | **0.38** | **0.52** | **0.47** | **0.10** | **0.46** |
|  | **E7** | **-** | **-** | **-** | **-** | **-** | **-** | **-** |
| **NDVI6** | **E1** | **0.19** | **0.21** | **0.09** | **0.34** | **0.12** | **0.11** | **0.85** |
|  | **E2** | **0.25** | **0.20** | **0.13** | **0.35** | **0.17** | **0.08** | **0.74** |
|  | **E3** | **-** | **-** | **-** | **-** | **-** | **-** | **-** |
|  | **E4** | **-** | **-** | **-** | **-** | **-** | **-** | **-** |
|  | **E5** | **0.16** | **0.18** | **0.09** | **0.35** | **0.19** | **0.09** | **0.67** |
|  | **E6** | **0.20** | **0.14** | **0.14** | **0.35** | **0.17** | **0.07** | **0.62** |
|  | **E7** | **-** | **-** | **-** | **-** | **-** | **-** | **-** |
| **CT1** | **E1** | **21.76** | **20.82** | **19.1** | **23.8** | **20.15** | **1.34** | **0.72** |
|  | **E2** | **22.70** | **21.50** | **19.4** | **21.8** | **20.62** | **1.30** | **0.40** |
|  | **E3** | **19.21** | **17.14** | **18.4** | **21.1** | **19.56** | **1.70** | **0.57** |
|  | **E4** | **22.36** | **20.15** | **20.4** | **24.1** | **21.56** | **1.46** | **0.77** |
|  | **E5** | **22.30** | **21.50** | **19** | **24.6** | **21.93** | **1.02** | **0.46** |
|  | **E6** | **21.35** | **22.21** | **20.7** | **23.2** | **25.74** | **1.02** | **0.32** |
|  | **E7** | **20.10** | **20.14** | **21.3** | **23.9** | **22.4** | **1.68** | **0.43** |
| **CT2** | **E1** | **21.4** | **21.23** | **22.3** | **25.7** | **23.62** | **0.97** | **0.65** |
|  | **E2** | **22.60** | **22.10** | **21.2** | **25.1** | **22.41** | **0.65** | **0.65** |
|  | **E3** | **23.25** | **21.14** | **19.8** | **25.1** | **23.96** | **0.87** | **0.69** |
|  | **E4** | **25.56** | **21.56** | **22.8** | **27.1** | **24.9** | **1.21** | **0.69** |
|  | **E5** | **23.12** | **22.15** | **23.5** | **26.7** | **24.07** | **0.78** | **0.53** |
|  | **E6** | **25.15** | **26.30** | **22.5** | **25.6** | **23.47** | **1.04** | **0.56** |
|  | **E7** | **24.25** | **22.34** | **21.6** | **26.3** | **23.41** | **1.05** | **0.46** |
| **CT3** | **E1** | **22.43** | **21.15** | **22.3** | **26.4** | **25.46** | **2.65** | **0.65** |
|  | **E2** | **20.20** | **21.25** | **21.3** | **26.3** | **23.36** | **1.25** | **0.52** |
|  | **E3** | **27.12** | **24.23** | **21.8** | **27.9** | **25.7** | **0.69** | **0.89** |
|  | **E4** | **26.45** | **25.33** | **23.8** | **30.9** | **25.70** | **0.87** | **0.89** |
|  | **E5** | **24.23** | **22.25** | **22.12** | **27.6** | **25.01** | **0.74** | **0.82** |
|  | **E6** | **25.40** | **25.36** | **22.6** | **27.2** | **25.31** | **0.85** | **0.40** |
|  | **E7** | **27.30** | **23.14** | **22.5** | **7.3** | **24.5** | **1.01** | **0.53** |
| **CT4** | **E1** | **24.50** | **23.15** | **23.5** | **27.8** | **24.26** | **0.87** | **0.29** |
|  | **E2** | **23.42** | **23.52** | **22.5** | **27.3** | **23.45** | **0.92** | **0.43** |
|  | **E3** | **29.36** | **26.36** | **23.4** | **30.1** | **25.3** | **1.05** | **0.45** |
|  | **E4** | **27.13** | **29.52** | **24.56** | **31.24** | **26.34** | **0.78** | **0.46** |
|  | **E5** | **25.42** | **23.32** | **24.12** | **29** | **26.74** | **1.15** | **0.83** |
|  | **E6** | **27.01** | **28.53** | **24.54** | **29.3** | **26.8** | **1.20** | **0.53** |
|  | **E7** | **28.51** | **26.16** | **23.4** | **28.1** | **25.4** | **0.89** | **0.39** |
| **RWC** | **E1** | **63.33** | **72.42** | **51.82** | **96.22** | **77.46** | **8.16** | **0.74** |
| **CL** | **E1** | **5.67** | **9.94** | **4.60** | **12.14** | **8.36** | **1.42** | **0.75** |

Location code; E1-Delhi rainfed (2016-17), E2-Delhi irrigated (2016-17), E3-Indore restricted irrigation (2016-17), E4-Powarkheda rainfed (2016- 17), E5-( Delhi rainfed (2017-18), E6-Delhi irrigated (2017-18) and E7-Indore restricted irrigation (2017-18).

(-); indicate that observation is not recorded for that trait in respective environment

**Supplementary table S2. Phenotypic performance and distribution parameters for grain component traits of parents and BILs in seven environments.**

| **Trait** | **Env** | **HD2733** | **C306** | **BIL min** | **BIL max** | **BIL avg** | **SD** | **H^2^** |
| --- | --- | --- | --- | --- | --- | --- | --- | --- |
| **TGW** | **E1** | **45.85** | **51.21** | **33.6** | **63.4** | **48.77** | **6.31** | **0.89** |
|  | **E2** | **62.2** | **68.25** | **28.8** | **69.5** | **45.62** | **9.28** | **0.68** |
|  | **E3** | **46** | **55** | **28** | **67.1** | **49.28** | **5.98** | **0.47** |
|  | **E4** | **45.23** | **53.23** | **29.2** | **62.9** | **48.4** | **6.62** | **0.41** |
|  | **E5** | **43.23** | **50.25** | **33.22** | **63.9** | **48.7** | **4.39** | **0.89** |
|  | **E6** | **61.20** | **63.24** | **27.55** | **68.25** | **44.90** | **5.15** | **0.68** |
|  | **E7** | **48** | **56** | **37.8** | **60.5** | **50.41** | **4.35** | **0.69** |
| **GWPS** | **E1** | **16.23** | **21.14** | **5.12** | **30.41** | **18.45** | **2.57** | **0.79** |
|  | **E2** | **20.3** | **23.5** | **8.16** | **29.73** | **19.44** | **4.50** | **0.71** |
|  | **E3** | **16** | **19** | **9.9** | **34.9** | **19.77** | **4.48** | **0.53** |
|  | **E4** | **14.56** | **18.2** | **4** | **24.82** | **15.53** | **2.77** | **0.74** |
|  | **E5** | **14** | **17** | **6.27** | **24.47** | **14.23** | **3.16** | **0.63** |
|  | **E6** | **19.5** | **21.3** | **7.59** | **24.48** | **18.27** | **3.81** | **0.81** |
|  | **E7** | **22** | **26** | **14** | **36** | **24.11** | **3.68** | **0.79** |
| **PH** | **E1** | **92** | **124.51** | **81.3** | **130.5** | **106.04** | **9.1** | **0.85** |
|  | **E2** | **99** | **141** | **90** | **149** | **118.53** | **13.11** | **0.85** |
|  | **E3** | **85** | **119** | **71** | **138** | **105.65** | **12.87** | **0.59** |
|  | **E4** | **77** | **95** | **58** | **130** | **89.59** | **12.74** | **0.39** |
|  | **E5** | **90** | **127** | **81** | **129** | **108.02** | **13.25** | **0.82** |
|  | **E6** | **91** | **132** | **85** | **139** | **115.05** | **11.33** | **0.74** |
|  | **E7** | **78** | **120** | **65** | **125** | **96.67** | **14.47** | **0.89** |
| **BIOMASS** | **E1** | **1253** | **1583** | **650** | **2150** | **1404.23** | **322** | **0.86** |
|  | **E2** | **1340** | **1610** | **100** | **2000** | **1291** | **348** | **0.71** |
|  | **E3** | **576** | **948** | **220** | **1460** | **848.27** | **392** | **0.58** |
|  | **E4** | **-** | **-** | **-** | **-** | **-** | **-** | **-** |
|  | **E5** | **1053** | **1685** | **850** | **1980** | **1130** | **272** | **0.83** |
|  | **E6** | **1208** | **1625** | **930** | **2150** | **1510.28** | **266** | **0.84** |
|  | **E7** | **724** | **1128** | **220** | **1460** | **848.27** | **198** |  |
| **HI** | **E1** | **0.37** | **0.41** | **0.08** | **0.48** | **0.37** | **0.012** | **0.46** |
|  | **E2** | **0.36** | **0.29** | **0.06** | **0.49** | **0.35** | **0.01** | **0.61** |
|  | **E3** | **0.33** | **0.31** | **0.03** | **0.48** | **0.31** | **0.10** | **0.65** |
|  | **E4** | **-** | **-** | **-** | **-** | **-** | **-** | **-** |
|  | **E5** | **0.36** | **0.31** | **0.11** | **0.46** | **0.34** | **0.05** | **0.78** |
|  | **E6** | **0.44** | **0.34** | **0.12** | **0.51** | **0.39** | **0.04** | **0.85** |
|  | **E7** | **0.38** | **0.35** | **0.12** | **0.52** | **0.37** | **0.05** | **0.70** |
| **GY** | **E1** | **441** | **612** | **115** | **765** | **442.67** | **125** | **0.79** |
|  | **E2** | **545** | **554** | **92** | **788** | **482.66** | **116** | **0.76** |
|  | **E3** | **191** | **299** | **23** | **567** | **274.82** | **95.1** | **0.86** |
|  | **E4** | **-** | **-** | **-** | **-** | **-** | **-** | **-** |
|  | **E5** | **389** | **524** | **180** | **695** | **467.59** | **93** | **0.69** |
|  | **E6** | **536** | **562** | **150** | **690** | **493.08** | **100.92** | **0.66** |
|  | **E7** | **275** | **395** | **56** | **595** | **342.94** | **95** | **0.67** |
| **DH** | **E1** | **94** | **86** | **69** | **117** | **91.9** | **8.16** | **0.89** |
|  | **E2** | **98** | **94** | **84** | **114** | **98.65** | **7.9** | **0.87** |
|  | **E3** | **92** | **84** | **-** | **-** | **-** | **-** | **-** |
|  | **E4** | **91** | **82** | **-** | **-** | **-** | **-** | **-** |
|  | **E5** | **95** | **89** | **74** | **114** | **94.07** | **7.8** | **0.65** |
|  | **E6** | **99** | **94** | **66** | **115** | **98.65** | **8.5** | **0.89** |
|  | **E7** | **91** | **84** | **-** | **-** | **-** | **-** |  |
| **SL** | **E1** | **9.62** | **12.54** | **5.1** | **15.3** | **10.84** | **1.11** | **0.68** |
|  | **E4** | **7.50** | **9.31** | **4.5** | **9.5** | **7.08** | **0.86** | **0.61** |
| **AL** | **E1** | **4.56** | **7.76** | **2.16** | **8.12** | **5.7** | **1.08** | **0.45** |
| **FLA** | **E1** | **23.36** | **26.42** | **16.32** | **43.45** | **25.42** | **4.61** | **0.62** |

Locationcode; E1-Delhi rainfed (2016-17), E2-Delhi irrigated (2016-17), E3-Indore restricted irrigation (2016-17), E4-Powarkheda rainfed (2016- 17), E5-( Delhi rainfed (2017-18), E6-Delhi irrigated (2017-18) and E7-Indore restricted irrigation (2017-18).

(-); indicate that observation is not recorded for that trait in respective environment

|  | **GY** | **DH** | **NDVI-1** | **NDVI-2** | **NDVI-3** | **NDVI-4** | **NDVI-5** | **RWC** | **CT-1** | **CT-2** | **CT-3** | **CT-4** | **GWPS** | **PH** | **BIOMASS** | **SPAD** | **TGW** |
| --- | --- | --- | --- | --- | --- | --- | --- | --- | --- | --- | --- | --- | --- | --- | --- | --- | --- |
| **GY** | **1** |  |  |  |  |  |  |  |  |  |  |  |  |  |  |  |  |
| **DH** | **0.052** | **1** |  |  |  |  |  |  |  |  |  |  |  |  |  |  |  |
| **NDVI-1** | **0.031** | **0.014** | **1** |  |  |  |  |  |  |  |  |  |  |  |  |  |  |
| **NDVI-2** | **0.238*** | **0.122** | **0.276*** | **1** |  |  |  |  |  |  |  |  |  |  |  |  |  |
| **NDVI-3** | **0.375**** | **0.217*** | **0.125*** | **0.344*** | **1** |  |  |  |  |  |  |  |  |  |  |  |  |
| **NDVI-4** | **0.126*** | **0.451*** | **0.326** | **0.365** | **0.263*** | **1** |  |  |  |  |  |  |  |  |  |  |  |
| **NDVI-5** | **0.327**** | **0.356** | **0.425** | **0.256** | **0.357** | **0.457** | **1** |  |  |  |  |  |  |  |  |  |  |
| **RWC** | **0.216*** | **-0.05** | **0.012** | **0.115*** | **0.056** | **0.324*** | **0.116*** | **1** |  |  |  |  |  |  |  |  |  |
| **CT-1** | **-0.152** | **-0.03** | **0.05** | **-0.251**** | **-0.125** | **-0.412*** | **-0.125** | **-0.242*** | **1** |  |  |  |  |  |  |  |  |
| **CT-2** | **-0.315**** | **0.01** | **0.157*** | **-0.122*** | **-0.223** | **-0.332*** | **-0.342** | **-0.233*** | **0.342*** | **1** |  |  |  |  |  |  |  |
| **CT-3** | **-0.412*** | **-0.127** | **-0.036** | **-0.256** | **-0.241** | **-0.051** | **-0.126** | **-0.124** | **0.126*** | **0.053** | **1** |  |  |  |  |  |  |
| **CT-4** | **-0.256**** | **0.036** | **--0.235** | **0.063** | **-0.356** | **-0.263** | **--0.145** | **-0.320*** | **0.148*** | **0.421*** | **0.217*** | **1** |  |  |  |  |  |
| **GWS** | **0.385**** | **-0.245*** | **0.07** | **0.148*** | **0.233*** | **0.148**** | **0.252**** | **0.115**** | **0.041** | **-0.396*** | **-0.231*** | **-0.304*** | **1** |  |  |  |  |
| **PH** | **0.071** | **0.236*** | **0.012** | **0.025** | **0.04** | **0.045** | **0.012** | **0.045** | **0.152** | **0.115** | **0.124** | **0.241*** | **0.221*** | **1** |  |  |  |
| **BIOMASS** | **0.373**** | **0.212*** | **0.234*** | **0.104** | **0.365*** | **0.324** | **0.234*** | **0.121** | **-0.124** | **0.405**** | **0.263*** | **0.157*** | **0.089** | **0.331**** | **1** |  |  |
| **SPAD** | **0.371**** | **0.131** | **0.557**** | **0.432*** | **0.452*** | **0.441*** | **0.557**** | **0.234*** | **0.051** | **0.045** | **-0.215*** | **-0.321*** | **0.339**** | **0.103*** | **0.373**** | **1** |  |
| **TGW** | **0.324**** | **-0.243*** | **0.331**** | **0.212*** | **0.101*** | **0.324*** | **0.331**** | **0.245**** | **-0.256*** | **-0.428**** | **-0.235**** | **-0.252*** | **0.242**** | **0.127**** | **0.245**** | **0.457**** | **1** |

**Supplementary table S3. Phenotypic correlation among physiological and yield component traits in the BILs population.**

**Supplementary table S4a. ANOVA for physiological traits in seven environments.**

| **Source** | **DF** | **NDVI1** | **NDVI2** | **NDVI3** | **NDVI4** | **Chlorophyll content** | **CT1** | **CT2** | **CT3** | **CT4** |
| --- | --- | --- | --- | --- | --- | --- | --- | --- | --- | --- |
| **Gen.** | 182 | 0.0035* | 0.0110* | 0.0161** | 0.0200** | 23.78048 | 21.625* | 778.283** | 26.420* | 24.572* |
| **Rep.** | 1 | 0.0144 | 0.0010 | 0.0014 | 0.0071 | 0.11981*, | 9.277 | 60.910 | 21.736 | 4.737 |
| **Env.** | 6 | 2.9461* | 7.1445 | 1.2372** | 9.7583** | 6617.892** | 7826.206* | 10124.79** | 7829.589** | 6508.68** |
| **Env. × Gen.** | 1092 | 0.0026* | 0.0048 | 0.0068** | 0.0098** | 11.50487* | 2.21902 | 5601.15* | 221.854* | 177.732* |

**Supplementary table S4b. ANOVA for yield component traits in seven environments.**

| **source** | **DF** | **PH** | **TGW** | **Biomass** | **Harvest index** | **GWPS** | **Grain yield** |
| --- | --- | --- | --- | --- | --- | --- | --- |
| **Gen.** | 1 | 320.0216** | 117.7381** | 135832.5** | 1058.86* | 26.4992** | 29876.68** |
| **Rep.** | 6 | 8.3640* | 6.10870 | 94.5 | 181.73* | 6.371 | 761.67 |
| **Env.** | 182 | 36983.624** | 703.6436** | 3140159.2** | 46897.80** | 4211.29** | 2826759.56** |
| **Env. × Gen.** | 1092 | 185.6661** | 50.64331** | 74139.4** | 1053.01* | 24.7300** | 13909.69** |

Note: **significance at the 0.01 level; Gen, genotype; Env. environments; Rep. replication

**Supplementary table S5. Distribution of markers and marker density across chromosomes in the linkage map developed in BILs of HD2733/2*C306.**

| **Chromosome** | **No. of markers** | **Map distance (cM)** | **Map density (cM/marker)** |
| --- | --- | --- | --- |
| **1A** | **50** | **139.07** | **2.78** |
| **1B** | **40** | **112.21** | **2.80** |
| **1D** | **23** | **230.97** | **10.04** |
| **2A** | **60** | **393.24** | **6.55** |
| **2B** | **66** | **220.92** | **3.33** |
| **2D** | **11** | **86.74** | **7.88** |
| **3A** | **48** | **264.78** | **5.51** |
| **3B** | **58** | **280.48** | **4.84** |
| **3D** | **17** | **118.87** | **6.99** |
| **4A** | **17** | **236.75** | **13.92** |
| **4B** | **30** | **175.81** | **5.86** |
| **4D** | **12** | **113.58** | **9.47** |
| **5A** | **53** | **303.46** | **5.72** |
| **5B** | **63** | **294.03** | **4.66** |
| **5D** | **15** | **326.30** | **21.75** |
| **6A** | **37** | **191.40** | **5.17** |
| **6B** | **41** | **287.15** | **7.00** |
| **6D** | **13** | **250.05** | **19.23** |
| **7A** | **69** | **288.10** | **4.17** |
| **7B** | **60** | **280.94** | **4.68** |
| **7D** | **8** | **105.3** | **13.16** |
| **A Genome** | **334** | **1816.8** | **73.82** |
| **B Genome** | **358** | **151.54** | **33.1** |
| **D Genome** | **99** | **1231.81** | **88.52** |
| **Total** | **791** | **4700.15** | **5.93** |

**Supplementary table S6****. A summary of co-localization of functional genes in the QTL confidence regions.**

| Trait | Env | QTL | Genetic location (cM) | physical location | No. of candidate genes within physical location of QTL | Candidate gene description |
| --- | --- | --- | --- | --- | --- | --- |
| TGW | E1 | *QTgw.iari_7A* | 135 | 515006529-545545628 | 4 | ATP-dependent 6-phosphofructokinase, Coatomer subunit beta, Acyl-[acyl-carrier-protein, Fatty acyl-CoA reductase, |
| TGW | E5 | *QTgw.iari_2A* | 84 | 729175029-734910228 | 6 | Protein phosphatase 2C, Plasma membrane ATPase, NADPH--cytochrome P450 reductase, auxin responsive protein, Golgin candidate 2, Vacuolar cation/proton exchanger |
| GY | E5 | *QYld.iari_2A* | 43 | 760619493-771506671 | 6 | Probable magnesium transporter, WAMP-3.1, antimicrobial peptide, Protein STAY-GREEN LIKE, chloroplastic, DDB1- and CUL4-associated factor homolog 1, Amidase 1, Peroxidase |
| GY | E2 | *QYld.iari_2B* | 155 | 655607120-660694870 | 5 | Group II catalytic intron, Phospholipid scramblase, Secretory carrier-associated membrane protein, U1 spliceosomal RNA, Vacuolar cation/proton exchanger |
| HI | E1 | *QHi.iari_2A* | 56 | 751609428-758420888 | 4 | Exocyst subunit Exo70 family protein, Triticain beta, Serine/threonine-protein kinase, Auxin response factor, |
| GWPS | E6 | *QGwps.iari_1A* | 23 | 12303846-12369432 | 1 | Probable serine/threonine-protein kinase WNK3 |
| RWC, NDVI-6 | E1 | *QRwc.iari_7A* | 143 | 645028871-652693872 | 6 | ATP-dependent 6-phosphofructokinase, Coatomer subunit beta, Acyl-[acyl-carrier-protein, Fatty acyl-CoA reductase, 3-ketoacyl-CoA synthase, Profilin |
| NDVI-3, NDVI-4, chl content | E2 | *QNdvi3.iari_5A* | 221 | 556006342-561109769 | 10 | Histone H4 variant TH011, UTP--glucose-1-phosphate uridylyltransferase, Nudix hydrolase 23, chloroplastic  , Mitochondrial phosphate carrier protein 1, mitochondrial, QPT , Sulfotransferase, Carboxyl-terminal-processing peptidase 2, chloroplastic, Signal peptidase complex subunit 3, SYCO ARATH, tRNA-Ser for anticodon AGA, |
| chl content | E1 | *QChl.iari_2A* | 61 | 751609428-752277545 | 4 | Phosphoglycerate kinase, Actin-related protein 8, Ubiquitin-conjugating enzyme, Group II catalytic intron |
| chl content | E2 | *QChl.iari_4A* | 207 | 29793110-46123526 | 21 | Ubiquitin carboxyl-terminal hydrolase 26 , Homeodomain-leucine zipper transcription factor, Small nucleolar RNA SNORD25, Undecaprenyl pyrophosphate synthetase family protein,30S ribosomal protein S10, chloroplastic,CASP-like protein, RNA cytidine acetyltransferase, Probable ribosome biogenesis protein,Cytochrome b561 and DOMON domain-containing protein, 40S ribosomal protein S3a, Protein NPGR1, Elongator complex protein 5, Adenylyl-sulfate kinase, Alpha/beta hydrolase, Strigolactone receptor, Strigolactone perception, Reguration of shoot branchig, Calcium-transporting ATPase,5'-3' exonuclease family protein,MAPR4, Sulfhydryl oxidase, CASP-like protein, Protein RBL, Integral membrane protein, Dirigent protein, S-acyltransferase, Cysteine proteinase inhibitor, Hexosyltransferase |
| PH | E1 | *QPh.iari_4B-3* | 107 | 24554735-28959979 | 11 | Lysine--tRNA ligase, Proteasome subunit beta type, Protein yippee-like, ATCES1, Pescadillo homolog, Adenylosuccinate synthetase, chloroplastic, Lipoxygenase, Phosphatidylinositol 4-phosphate 5-kinase, Glycosyltransferase, Putative eukaryotic peptide chain release factor subunit 1-3, SET domain group 37 |


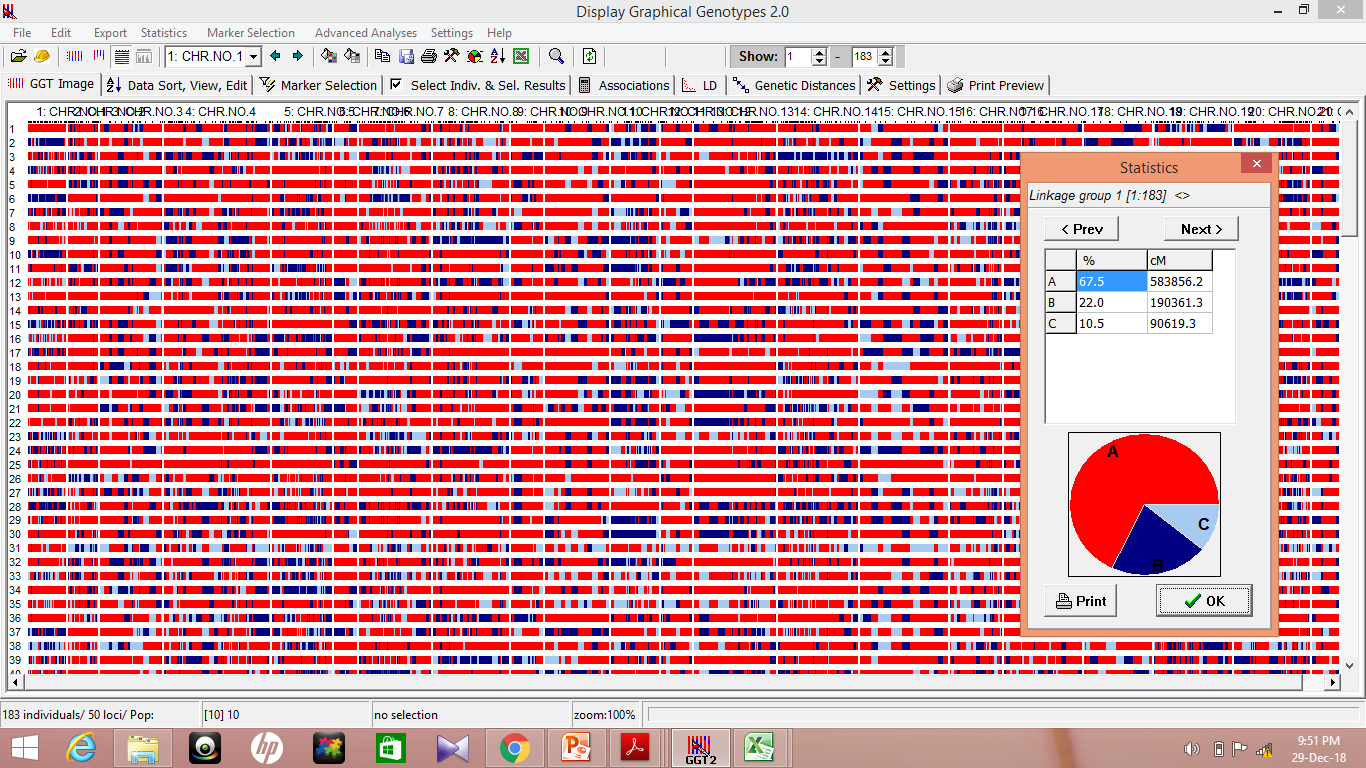


**Supplementary Figure S1. Graphical genotypes of 183 HD2733/2*C306 BILs genotypes using 706 SNP and 86 SSRs in BCIF6 (red- HD2733, dark blue-C306 and sky blue-heterozygotes)**
